# Health seeking for chronic lung disease in central Malawi: Adapting existing models using insights from a qualitative study

**DOI:** 10.1371/journal.pone.0208188

**Published:** 2018-12-17

**Authors:** Sepeedeh Saleh, Grace Bongololo, Hastings Banda, Rachael Thomson, Berthe Stenberg, Bertie Squire, Rachel Tolhurst, Laura Dean

**Affiliations:** 1 Liverpool School of Tropical Medicine, Pembroke Place, Liverpool, United Kingdom; 2 Research for Equity and Community Health (REACH) Trust, Lilongwe, Malawi; 3 LHL International Tuberculosis Foundation, Oslo, Norway; University of Calcutta, INDIA

## Abstract

**Background:**

Chronic lung diseases contribute to the growing non-communicable disease (NCD) burden and are increasing, particularly in many low and middle-income countries (LMIC) in sub-Saharan African. Early engagement with health systems in chronic lung disease management is critical to maintain quality of life and prevent further damage. Our study sought to understand health seeking behaviour in relation to chronic lung disease and TB in a rural district in Malawi.

**Methods:**

Qualitative data was collected between March-May 2015, exploring patterns of health seeking for lung disease amongst residents of two districts in rural Malawi. Participants included those with and without lung disease, health workers and village leaders. Participants with a history of TB were included in the sample due to similarities in clinical presentation and in view of potential to cause long-term damage to lung tissue.

**Results:**

Our findings are ordered around a specific model of health seeking devised by adapting previous models. The model and findings span three broad areas that were found to influence health seeking: understandings of health and disease which shaped whether, when and where to seek care; the care seeking decision which was influenced by social and structural factors; and the care seeking experience which impacted future care decisions creating ‘feedback loops’.

**Discussion:**

Efforts to improve effective and accessible healthcare provision for chronic lung disease need to address all the determinants of health seeking behaviour identified. This may include: enhancing the structural and financial accessibility of health services, through the strengthening of community linkages; improving communication between formal health providers, patients and communities around symptoms, diagnosis and management of chronic lung diseases; and improving the quality of diagnostic and management services through the strengthening of health systems ‘hardware’ (equipment availability) and ‘software’ (development of trusting and respectful relationships between providers and patients).

## Introduction

Globally, the burden of non-communicable diseases (NCDs) is increasing. In sub-Saharan Africa, between 1990 and 2010 deaths from NCDs rose by 46% [1, 2]. This shift in disease burdens presents a new challenge for health systems, as they need to adapt to meet the chronic long-term care needs of their populations, whilst continuing to deliver curative health interventions for acute disease. Chronic lung diseases, including asthma, chronic obstructive pulmonary disease (COPD) and bronchiectasis contribute to the growing NCD burden, with approximately 235 and 250 million people living with asthma and COPD worldwide [3, 4]. Chronic lung diseases are thought to be increasing in many low and middle-income countries (LMICs) in sub-Saharan Africa, including Malawi, and are associated with smoking epidemics, indoor and outdoor air pollution and increasing life expectancy [5]. Mortality and morbidity associated with chronic lung diseases is particularly high in LMICs, which account for over 90% of COPD deaths globally [4]. Such global disparities in outcomes associated with chronic lung diseases are likely to be due in part to poor healthcare access resulting in delays in care seeking, diagnosis and treatment. Such delays, coupled with the persistently high rates of infectious lung diseases such as TB, often result in a need for chronic care for people affected by communicable lung disease due to permanent organ damage. Consequently, health systems are left with a double burden in managing morbidity associated with non-communicable and communicable lung disease in these settings [6].

Early engagement with health systems in chronic lung disease management, as well as for infectious diseases such as TB, is critical to reduce the frequency of chronic disease exacerbations and prevent further permanent damage to the lungs, helping to maintain quality of life in the longer term. Strengthening of health systems to ensure they are prepared to diagnose, treat and manage chronic lung diseases when people present at health facilities is also essential. Whilst there is no lack of research on health seeking in general, very little of the literature describes health seeking for chronic disease, specifically, chronic lung diseases, particularly in a landscape characterised by medical pluralism and in low-income settings such as Malawi. In addition, despite the growing evidence base regarding under-diagnosis of chronic lung diseases in LMICs such as Malawi [7, 8] little is currently known about diagnosis and treatment as part of the health seeking pathway.

There are key differences between health seeking in chronic disease and health seeking for one-off, curable illnesses. Chronic diseases involve recurrent episodes of care seeking for similar symptom complexes, thus increasing the significance of each care-seeking episode in terms of its repercussions on future health perceptions. In working towards strengthening health systems to ensure early and equitable access to effective diagnosis and management, it is therefore important to first understand patterns of health seeking in patients with respiratory symptoms.

## Study aims and objectives

Our study sought to understand current health seeking behaviour in relation to chronic lung disease and TB in a rural district in Malawi. Participants with a history of TB were included in the sample in addition to those with chronic lung disease symptoms, due to the similarities in clinical presentation, often causing confusion in diagnosis, and in view of the potential of both to cause long-term damage to lung tissue if not addressed. Specific objectives were: 1) to describe how various coexisting understandings of illness and disease, as well as other factors, can shape health seeking for chronic lung disease in the context of pluralistic health systems in central Malawi; 2) to explore the characteristics of health seeking pathways for chronic lung disease and TB in central Malawi; 3) to make recommendations for appropriate health systems responses to support people living with chronic lung disease symptoms in central Malawi in accessing health care.

## Methods

### Study sites

The study was part of the pre-intervention stage of the Triage Plus trial, Phase II (trial ID PACTR201411000910192) which aims to improve referral, diagnosis and follow-up for chronic lung disease (COPD, Bronchiectasis and Asthma) and TB in Malawi by assessing the potential of engaging community health providers to assist in the diagnostic process of chronic lung disease and TB. It also focuses on embedding at facility level the World Health Organization’s Practical Approach to Lung health plus (PAL+) guidelines, which advocate a specific approach to these patients based on identifying and managing groups of signs and symptoms. The purpose of the pre-intervention phase was to better understand the current context and health seeking in relation to chronic lung disease and TB at community level in Malawi. Kasungu district was selected as the study site for the qualitative pre-intervention phase as it was similar in context (geography, population etc.) to the main Triage trial sites of Dowa and Ntchisi. Kasungu district is located in the central region of Malawi covering an area of 7,878km^2^, with a population of 892,523, the majority of whom are from the Chewa tribe [9]. Data were collected between March and May 2015. Within Kasungu district two health facilities were purposively selected to achieve maximum variation in context based on population size, ethnicity and geography. Both facilities were public facilities that provide treatment free at point of delivery. The shared population of these catchment areas was almost 88 000.

### Study design

This study utilised qualitative research methods as listed in Tables 1 and 2. Data collection included 9 separate Focus Group Discussions (FGDs) with male and female community members (4), informal health providers (2), health surveillance assistants (1) and village health committees (2); 5 key informant interviews (KIIs) with officers in charge/medical assistants and village chiefs; and 14 in-depth interviews with patients identified as having chronic cough or a diagnosis of TB, asthma, COPD or bronchiectasis (as recorded in health passports). Researchers took an inclusive approach to participant selection: given the under-representation of people living with disabilities in studies of this type we specifically sought opinions from this sub-population. Six local field staff (three male and three female) completed the data collection under the co-ordination of GB. Interviews with community members and FGDs were conducted in Chichewa and KIIs with medical staff, in English.

**Table 1:** Key informants and focus group discussion participants.

**Focus Group Discussions (number of participants per group)**	
Lay Women	2 (9), (10)
Lay Men	2 (9), (8)
Informal Health Providers	2 (8), (7)
Village Health Committees	2 (7), (8)
Health Surveillance Assistants	1 (9)
**Key Informant Interviews**	
Officer in Charge	2
Medical Assistant	1
Village Chief	1
Community Leader	1

**Table 2:** In-depth interview participant characteristics.

Disease/Symptom of Interest	Male	Female
Chronic Cough	3	4
Asthma	4	1
TB	1	2
Total	8	6 (including one participant with chronic cough and TB)

#### Key informant interviews

Key informant interviews were conducted as part of the community entry and permissions process and to gain a broad understanding of the health status (with specific reference to lung disease) of the communities within the selected facility catchment areas. Two types of key informant interview were undertaken with participants purposively selected due to their role within the health facility or their influential position within the community. At community level, the community chief and one additional community leader were interviewed to understand more about the community context in relation to health seeking, awareness of different lung health conditions, and the role of formal and informal health care providers in disease management. Within each facility, the officer in charge and medical assistant (where available) were interviewed to explore current practices in relation to the diagnosis and management of chronic lung disease within their facility, as well as exploration of other factors regarding community health seeking. All key informants were informed of the study before participation and a suitable time, date and location for their interview arranged.

#### Focus group discussions

Following key informant interviews, focus group discussions were conducted to elicit community perceptions of symptoms associated with chronic lung disease such as chronic cough, wheezing and breathlessness, as well as to explore understandings of health seeking and experiences in relation to these symptoms. FGDs were conducted separately with lay men and women from within the community, and participants were sampled for homogeneity in group composition with support from community leaders. Additional FGDs were also conducted with influential individuals and existing community groups. These included informal health providers (such as traditional healers and grocery shop owners); health surveillance assistants; and village health committee members. The focus of the questions in these groups was the same as in community FGDs, however, greater emphasis was placed on processes and practices in relation to interactions with chronic lung disease patients and the formal/informal health system. Prior to the FGD, the purpose of the study was explained to all participants, a suitable time and date for the activity agreed and informed consent taken.

#### In-depth interviews

Initially, a sampling frame of all individuals known to be living with chronic cough, asthma, or tuberculosis within each of the facility catchment areas was developed from out-patient department registers and chronic cough registers at each selected facility. Tuberculosis diagnoses were made at facility level using smear microscopy, with smear negative cases being diagnosed on a clinical basis. These followed guidance presented in the 2012 National TB Programme Manual in operation at the time of the study [10]. Chronic lung disease diagnoses, either recorded in registers or participants’ health passports, had previously been made by health workers, usually on a clinical basis in view of the lack of diagnostic equipment available at these rural centres and the uncommon nature of referral (and subsequent patient transport) to specialist central hospitals. Sampling frames were discussed with health surveillance assistants and any additional patients were added based on their knowledge of patients living with chronic cough in their catchment community. From the sampling frame, a purposive sample of participants was selected to maximise variation in age, gender, and diagnosis. No patients with a diagnosis of COPD or bronchiectasis were identified. This is likely to be due to a combination of unfamiliarity with these conditions amongst health practitioners and lack of access to spirometers and other diagnostic equipment to differentiate restrictive and obstructive lung disease, for instance [8]. Prior to interview, participants were visited in their homes to inform them about the study and to arrange a time and date for interview. In-depth interviews explored participants’ understanding of different chronic lung diseases and their symptoms (mainly chronic cough, breathlessness and wheezing); their experiences in relation to these diseases and symptoms; and their health seeking pathways, including diagnosis.

### Data analysis

Analysis was conducted using the framework approach [11]. An inductive coding framework was developed collaboratively between the research team. The framework was continually refined and adapted during the iterative analysis process. Data were coded by GB, SS, and SC and quality checked by LD. Once coded, charting was completed based on themes emerging within and between codes. Descriptive accounts of the findings were written by GB, LD and SS based on the charts developed. All authors then reviewed and refined descriptive accounts of the data. Within this process, similarities and differences were explored between the different data collection methods and participant accounts. Such interrogation of the descriptive account was used to generate an explanatory account of the data.

### Ethics statement

Ethical approval was obtained from the Liverpool School of Tropical Medicine, application reference 14.040, and the Malawi College of Medicine Research Ethics Committee (COMREC), P.07/13/1424. In addition, community sensitisation was completed, and approval obtained prior to data collection. Written consent was obtained in the local language from all participants.

## Results

Our results section is split into three broad areas based on our thematic analysis. The first area, stemming from focus group and interview data with key informants and community members with and without lung disease symptoms, focuses on ‘understandings and perceptions of chronic lung disease and TB’ and how this shaped whether, when and where to seek care. The second area, stemming from the same data, addresses the specific ‘care seeking decision’ linked to a specific illness episode; themes in this section focus the influence that individuals and wider society have in the care seeking decision. Lastly, drawing from all data, we explore ‘experiences of accessing healthcare’ for lung diseases, including perceptions of availability and quality of healthcare, lung disease diagnosis, and management. In discussions, participants spoke of their own experiences as well as those of friends and family. This is reflected in the discussion where relevant. As well as quotes presented in the results section, please also see the S1 Appendix for additional supporting data.

### Understandings and perceptions of chronic lung diseases and TB

In community members’ accounts, diseases were often identified as specific symptom complexes; thus, symptoms and diseases became interchangeable in illness narratives. There was an association between chronic cough or frequent coughing and TB, for instance, and links between breathlessness (also encompassing shortness of breath and difficulty breathing) and asthma. Some participants extended the definition of asthma to include *‘wheezing*, *like a chicken’* (Study site 1, interview with a patient with asthma diagnosis, 68 year old male) as a key symptom. Certain diseases were closely linked in participants’ consciousness to other diseases, for example, TB and HIV/AIDS.

*‘In short*, *we just say breathlessness (befu) instead of asthma (mphumu)’* (Study site 2, interview with a patient with chronic cough, 54 year old female)

COPD and bronchiectasis were rarely discussed, and participants were frequently unresponsive when asked about these diseases using the biomedical terms in Chichewa (as no alternative description was available), suggesting a lack of public awareness of these chronic diagnoses. Participants rarely described receiving information about chronic lung diseases, and suggestions of community information-giving around these issues were universally welcomed.

*‘I have never seen anyone coming to provide information about TB or asthma and also pneumonia*. *I have never seen anyone talking about that*. *Those that do come here they give advice on cholera*, *and malaria that is what I have seen’* (Study site 1, focus group with community men, 32 year old male)

#### Perceptions of disease severity shaping whether and when to seek care

Disease and symptom perceptions influenced participants’ health-seeking responses, with certain symptom-clusters (and associated diseases) being seen as more serious than others. Where diseases or symptoms were perceived to be ‘serious’, care seeking was quicker and more direct. Breathlessness (and asthma by association), for example, was generally seen as an urgent problem requiring immediate medical attention, usually at a formal healthcare facility.

*‘If it is breathlessness*, *we rush to hospital because a person may panic up to death … We don’t take time because is very dangerous*, *so if we delay we can lose that life*, *so we rush to the hospital’* (Study site 1, focus group with community women, 26 year old female)

When discussing health seeking for a chronic cough, which was frequently seen as a less serious symptom than breathlessness or other diseases such as Malaria, care seeking responses varied. Some participants felt that cough could be left to reach a critical point before they needed to seek care.

*‘There is a difference because when one has malaria people take him to hospital right away but for cough it takes time since he gets better and then sick again but when it gets very serious*, *it is when he goes to hospital’* (Study site 1, focus group with community women, 55 year old female)

Whilst some community members maintained that they *‘rush to the hospital’* (Study site 1, focus group with community women, 29 year old female) in response to cough symptoms, other pathways were also discussed, including use of traditional medicines and the purchase of drugs from the grocer. The perceived lack of urgency with respect to cough symptoms amongst some seemingly allowed space for varying health-seeking pathways, at times involving the sequential use of multiple options.

[Of people with chronic cough] *‘They delay [seeking care from the hospital] because they first seek help from traditional healers or buying medicine’* (Study site 1, focus group of health centre committee members, 60 year old male)

#### Perceptions of disease causation shaping where to seek care

Perceptions about the origins of symptoms impacted on decisions about where care was initially sought. The concept of ‘mdulo’, was a frequently cited example of this. Mdulo describes sickness or death of a person (child or adult) caused by the transgression of cultural taboos, usually related to sex, and was linked by several participants to cough symptoms, although it also has been described in the literature as having been linked to other illness types [12–14]. This led to a different care seeking pathway (involving a traditional healer) compared to care-seeking for illnesses attributed to biomedical causes, which would more often lead participants towards formal healthcare options.

*‘if a person has chronic cough*, *there are two ways*, *[either] he go(es) to hospital on the part of TB*, *but we also talked about mdulo*, *this one the hospital doesn’t heal it*, *that is why we go to (the) traditional healer* … *at the hospital they don’t deal with cultural beliefs’* (Study site 2, focus group with community men 35 year old male)

In practice, interpretations of disease causality were usually varied and not fixed. Participants often moved fluidly between seeking traditional, religious, and biomedical explanations for their symptoms in varying orders, with some narratives describing the parallel existence of both spiritual and biomedical components to a single illness.

*‘[If] we can think of someone we had a quarrel with*, *we cannot go to the hospital but we go to someone who can help us*, *they can be able to tell us who did it*, *but when we go to the hospital they cannot tell us that someone has made us to eat something*, *so we go to the hospital after being helped traditionally’* (Study site 1, focus group with community men, 26 year old male)

This co-existence of different belief systems amongst participants interacted in different ways to influence care seeking, as illustrated by a story told by a health care advisory committee member:

*‘There was a person who met a leopard*, *he was a pastor’s son*, *and he stayed in a tree but another man ran away*. *The one who stayed in the tree was eaten by the leopard*. *So [the moral of the story is that] the word of God says I help those that help themselves*: *if you went to the hospital you could have been given Panadol and I could have blessed you from there’* (Study site 2, focus group with health centre advisory committee members, 57 year old male)

Whilst this story illustrates how religious beliefs could support biomedical health seeking for some, interpretations of religion also sometimes acted as a deterrent for formal care seeking.

‘*Others go for prayers because their church does not allow them to take drugs*, *or the traditional ones’* (Study site 2, focus group with community women, 26 year old female)

The ability to call on alternative systems of understanding was frequently used to seek a cure when one form of care was seen to fail. In relation to chronic lung diseases for example, where formal healthcare was not able to provide a definitive diagnosis or cure (such as a positive TB test and subsequent treatment), participants then resorted to traditional healthcare options.

*‘People were saying there is no treatment at the hospital that heals asthma but traditional medicines are available that heals asthma’* (Study site 2, interview with a patient with asthma diagnosis, 33 year old male)

### The care seeking decision

#### Influences of other community members on health seeking

When discussing the people involved in care seeking decisions, participants most often cited family (and in particular parents) as contributing to and supporting these choices. Parents and grandparents were often reported to have passed down traditional beliefs regarding health and disease and consequently sometimes advised in favour of visiting traditional healers in times of illness.

*‘we just go to the forest to seek traditional medicine those that our parents taught us’* (Study site 1, focus group with community men, 27 year old male)

Where friends appeared in participants’ narratives around health seeking decisions they tended to be involved with sharing biomedical knowledge and encouraged participants to seek care from formal health facilities. Friends and other acquaintances were also cited as sources of practical help at times of illness: most often in terms of borrowing money or bicycles for transport to health facilities, as illustrated in the following section.

#### Wider influences on care seeking practices

Health seeking for lung disease was impacted by wider determinants, including economic costs, and factors relating to physical access to the health facility. Themes of distance (from the health facility) and infrastructure (allowing participants to reach the facility) emerged strongly from participants’ accounts of health seeking. Many participants, both in interviews and focus groups in both sites, stated that the health centres were far away and therefore difficult to access. Inadequate infrastructure such as a lack of *‘proper roads’ (*Study site 1, focus group with community men, 27-year-old male) constituted a further barrier, which interacted with seasonal factors such as rain, making journeys even more difficult, and at times leading people to seek alternative sources of help.

*‘When people think about the distance to reach the hospital they just go to the traditional healer first*, *so the distance also influences the decision to seek health care traditionally’* (Study site 1, focus group with community men, 32 year old male)

Transport costs also influenced whether and when care would (or could) be sought, and which type of provider was accessed. For example, hiring a bicycle or oxcart incurred costs, which were often prohibitive, particularly at times of scarcity. Participants sometimes described delaying care seeking, but often resorted to borrowing money or drawing in favours from others in the community to raise the necessary funds for transport.

*‘To borrow a bicycle from a friend it means one has to pay*, *but as you know to find money here*, *it goes with season*, *we wait for the rain’ (i*.*e*. *for crops to grow and to realise payment from the produce)* (Study site 1, focus group with community men, 27 year old male)

Opportunity costs of accessing care, for example missing out on valuable harvesting days for communities who relied at least partially on subsistence farming, acted as further delaying factors or deterrents to accessing formal healthcare for many. Help-seeking from traditional healers and grocers was not impacted in the same way, due largely to their proximity to households in the village.

*“The thing that makes people to [use traditional medicine]*, *it is because of lack of transport so as people are waiting to find the transport they use the traditional medicine ‘from our parents’” (*Study site 1, focus group with community men, 26 year old male)

Few participants described purchasing medications from the grocer, and those who did mainly bought painkillers, rather than more specific or curative treatment.

Alternative sources of healthcare such as traditional healers and grocers did not always replace more formal health seeking, but sometimes acted as a ‘first port of call’ at the time of initial sickness, forming one step in a sequence of health seeking events. This was especially the case when there were additional economic and physical barriers to accessing care at the health facility such as out of working hours or when the patient was thought to be too weak to travel.

*‘Here when we get ill urgently…and we want to [save] the life of our relative and have seen that the hospital is [far] away*, *we take that person to [the] traditional healer so that we at least make him recover a little and get energy to go to X hospital’* (Study site 1, focus group with community women, 37 year old female)

### Experiences of care seeking

#### Availability of healthcare

Having decided to seek care at a government facility, participants often encountered challenges relating to the availability of care. These included medication shortages (referred to as drug stock-outs), staff shortages and facility closure. Shortages of trained staff were frequently reported, leading to treatment delays or people being turned away without care, particularly in the evening or at night. A few patients who described attending public hospitals and clinics with symptoms generally perceived to be ‘urgent’, such as breathlessness and wheezing as seen in an asthma attack, gave more positive accounts of their experiences.

*‘They differentiate asthma with other diseases because it is very difficult*, *they make sure that if the person has an attack he/she does not stay on the line but is treated instantly so that the person can get better because one can die easily with asthma but when going to take the [asthma] drugs I stay on the line like anyone else’* (Study site 2, interview with a patient with asthma diagnosis, 75 year old female)

Inability to access care at government facilities had cost implications for participants, who often turned to private medical facilities to pay for treatment or drugs.

*‘As we know the private [hospitals] are doing business so they make sure that they always have medical supplies in their stock and when people go there they find treatment’* (Study site 1, interview with the health worker in charge at the health centre, 30 -year old male)

This was exacerbated for participants with chronic lung diseases, who experienced multiple episodes of recurrent illness, both prior to and following diagnosis, and who were often reliant on regular medications.

Many participants cited these difficulties as factors in describing how they made future health-seeking decisions. Such decisions were influenced by their own experience as well as those of friends and family.

*‘I went to the hospital with my child I carried him on my back*, *I started off at around past three and arrived there around past 4 pm*, *they rejected me until the child died on my back and from that time I said I will never come to this hospital’* (Study site 2, focus group with community men, 35 year old male)

#### Diagnosis of lung disease

On presenting to a formal health facility with breathlessness or coughing, the first response in most cases appeared to be to test for TB: a common cause of respiratory symptoms in this setting. Where participants tested negative for TB, some described having the test repeated, to ensure that a diagnosis of TB had not been wrongly ruled out. Another relatively common pathway for those presenting with respiratory symptoms but testing negative for TB—and sometimes without TB testing—was the prescription of medications by health providers at the local clinic or doctors at the hospital (either antibiotics- ‘Bactrim’- or simple painkillers- ‘Panadol’), for presumed pneumonia: another common cause of respiratory symptoms in Malawi.

*‘In most cases the person comes to hospital and we give him Bactrim*, *Amoxil and if we see that the person is not responding to the antibiotics then we start thinking about TB*, *getting sputum from him’* (Study site 2, interview with the health worker in charge at the health centre, 42 year old male)

In some cases, conditions such as asthma were subsequently diagnosed following a negative TB test, and often after other medications had failed to improve symptoms. These diagnoses invariably followed referral to a central hospital. Many participants described delays at this stage, often for long periods of time, with repeated visits to a health facility being made before accessing a diagnosis or effective treatment.

*‘I was going to hospital more frequently until my health passport book was filled with cough diagnosis…I would say it took close to two years for me to get a diagnosis of asthma’* (Study site 2, interview with a patient with asthma diagnosis, 33 year old male).

Health workers frequently tried various medications rather than referring patients to the central hospital due to limited recognition of chronic lung conditions such as asthma, COPD and bronchiectasis. Most health workers described an unmet need for education and training regarding diseases such as COPD and asthma amongst primary health centre staff.

*‘On lung health there are a lot of things and we need to know them*. *We have talked about bronchitis which we said it is difficult to diagnose it at health centre level*. *It might be possible to diagnose it at earlier stage maybe it’s just lack of knowledge in me*, *I would like to know more about that’* (Study site 2, interview with a health worker in charge at the health centre, 42 year old male).

Following referral, challenges related to physical distance and transport re-emerged for patients when trying to access the hospital, exacerbating delays to chronic lung disease diagnosis for many participants.

#### Communication of lung disease diagnosis

There was little evidence of effective communication around chronic lung disease diagnoses and their repercussions for the patient, although a few asthma patients did name their condition and describe having been informed by doctors that it was not curable. COPD was not recognised in FGDs and no diagnosed cases could be identified from the register. Many of those with ‘chronic cough’ appeared to be still searching for a diagnosis. This contrasted with TB for instance, where participants frequently quoted positive test results.

*‘they told me that they did not find anything in your sputum*, *we were expecting that we will find TB but it is not there*. *And the main problem I am facing now is the lack of a “sense of test” (diagnostic test)’* (Study site 2, interview with a patient with chronic cough, 56 year old male)

Informal health providers, who could potentially be central to this process of communication, also commonly voiced a wish for more education and training on respiratory conditions, including chronic lung diseases.

*‘There should be groups that will be trained and those groups should be teaching other people about lung health*, *TB and asthma*, *how we can prevent them and how to deal with them in general’* (Study site 2, focus group with informal health providers, 54 year old female).

#### Treatment for chronic lung diseases

In terms of treatment of lung conditions beyond TB, health workers’ and patients’ knowledge levels varied by condition. Some health workers named specific asthma medications such as salbutamol and aminophylline, used both in prevention and treatment of acute exacerbations: others described giving painkillers, or using traditional remedies. A few asthmatic participants could name their medications while others described the tablets or inhalers they had been prescribed. Despite the lack of a definitive cure, many participants with asthma diagnoses were committed to their treatment, reporting its positive effects on controlling their symptoms. There were reports by patients of medication underuse, usually in stated attempts to make the medications provided last longer. This may possibly also be related to inadequate communication surrounding the nature of chronic disease and the need for lifelong treatment in some cases: participants often ended interviews by asking about potential cures for asthma. Neither patients nor healthcare staff mentioned COPD management, again suggesting a lack of knowledge surrounding the condition.

People who had been diagnosed with TB seemed, overall, accepting of their treatment, acknowledging its negative repercussions if untreated, and the curative potential of the medications.

*‘They were giving me tablets*, *I was taking them in the morning and evening until the cough left me because I followed the procedure told by the doctor and my other friend who did not listen to the advice were just throwing the drugs on the window because the drugs are very bitter … I am saying my friend died because of not understanding the doctor’* (Study site 2, interview with a patient with history of TB, 51 year old male).

#### Experiences of service quality

‘Trustworthiness’ emerged as a common theme in participants’ perceptions of treatment quality at various facilities. Regarding private healthcare facilities, while the absence of drug stock-outs and staff shortages was often positively commented on, many accounts reflected an overwhelming perception that these hospitals existed *‘to maximize profit’* (Study site 2, interview with the health worker in charge at the health centre, 42 year old male). Participants frequently described injection solutions being diluted to stretch to more patients, wrong dosages being given, and unnecessary injections being administered without examination or diagnosis, with the emphasis on payment. Grocers were framed in similar terms, with stories of people being given expired drugs and incorrect doses of medications.

*‘some drugs people buy from the grocery* …*are adding diseases to the child because the drugs have already expired*. *At hospital*, *they will give the right dosage to the child but from the grocery you just give him 4 or 2 tablets which can be an overdose’* (Study site 1, focus group with informal health practitioners, 40 year old male).

This ‘trustworthiness’ related also to a sense of provider honesty in further management of illnesses, with participants valuing onward referral where necessary. Opinions on traditional healers in this regard were mixed, with some descriptions of healers recognising their limits and referring patients to health centres, but other participants denying this.

*‘At the hospital they know whether it is the problem of the lungs they will say here we cannot manage go to facility X and if they see that they cannot do it they will refer you to hospital X*, *yet at the traditional healers they will just hold you in order for death to find you there*, *so actually people go to hospital’* (Study site 1, focus group with informal health practitioners, 40 year old male)

Expectations around diagnosis and treatment quality were also important in shaping participants’ perceptions of health service quality. This was evident in comparisons of private and public hospitals: participants valued efforts by doctors at the public hospital to examine patients and diagnose the cause of the problem (usually in terms of TB diagnosis), whereas private hospitals were seen as being more concerned with administering treatment. Health surveillance assistants working at the clinic revealed the value many patients placed on receiving certain types of treatments, in particular those seen to be more powerful (such as medications given in injection form).

*‘Most villagers are attracted when they are provided with more drugs or injections*. *They believe injection is the best treatment one can get’* (Study site 1, interview with the health worker in charge at the health centre, 30 year old male)

Attitudes of staff at health facilities were a further contributing factor to participants’ perceptions of the services they accessed. Accounts of treatment by staff at public facilities were mixed, with many patients claiming that they were *‘well received’*, but other descriptions involving rudeness and even discrimination, particularly in the case of people with disabilities. There was a suggestion by some, both patients and staff, that such negative attitudes could be linked to understaffing and consequent pressures on existing staff.

*‘There when they write the drugs for us*, *sometimes it is okay but other times he is rude*, *maybe because when someone is tired they get rude’* (Study site 2, interview with a patient with HIV diagnosis and chronic cough, 54 year old female)

Descriptions of poor staff conduct towards patients attending health clinics were often contrasted with experiences of private hospitals, where participants described receiving a more positive reception.

*‘At private [hospitals] I have never heard [from] anyone that they were not treated well*, *it does not happen*: *at the private [hospital] they treat and help a person very well’* (Study site 1, focus group with community men, 32 year old male)

Here again the experiences of friends and family, as well as personal experiences of health service quality, shaped perceptions, and consequently decisions regarding future health seeking. Social influences were woven throughout the process.

## Discussion

### Key results

Data from interviews and focus groups with community members in our study depicted how initial health seeking decisions were shaped by systems of understanding around health and illness that predated the illness episode in question, as well as by more immediate factors such as perceptions of disease severity. Further factors affecting these decisions included social influences and structural factors: where barriers existed precluding one source of care—whether economic, geographical, or logistical—other sources were likely to take precedence. The data also reflected the role of health-seeking experiences in colouring future care seeking decisions for participants with respiratory symptoms or chronic lung disease diagnoses. Poor experiences negatively influenced perceptions of the service in question. Data from focus groups and interviews with health professionals confirmed these systemic pressures and revealed more about professionals’ knowledge surrounding chronic lung diseases. Diagnosis, communication, and management of chronic lung diseases were found to be areas of weakness for formal health services in Malawi. These findings suggest that better understanding of chronic lung disease amongst Malawian communities could increase early presentation and management of such diseases in this population. This, however, would depend on concurrent attention to the existing barriers to accessing and delivering these sources of care.

### A model of health seeking for chronic lung disease symptoms in Malawi

**Fig 1:**
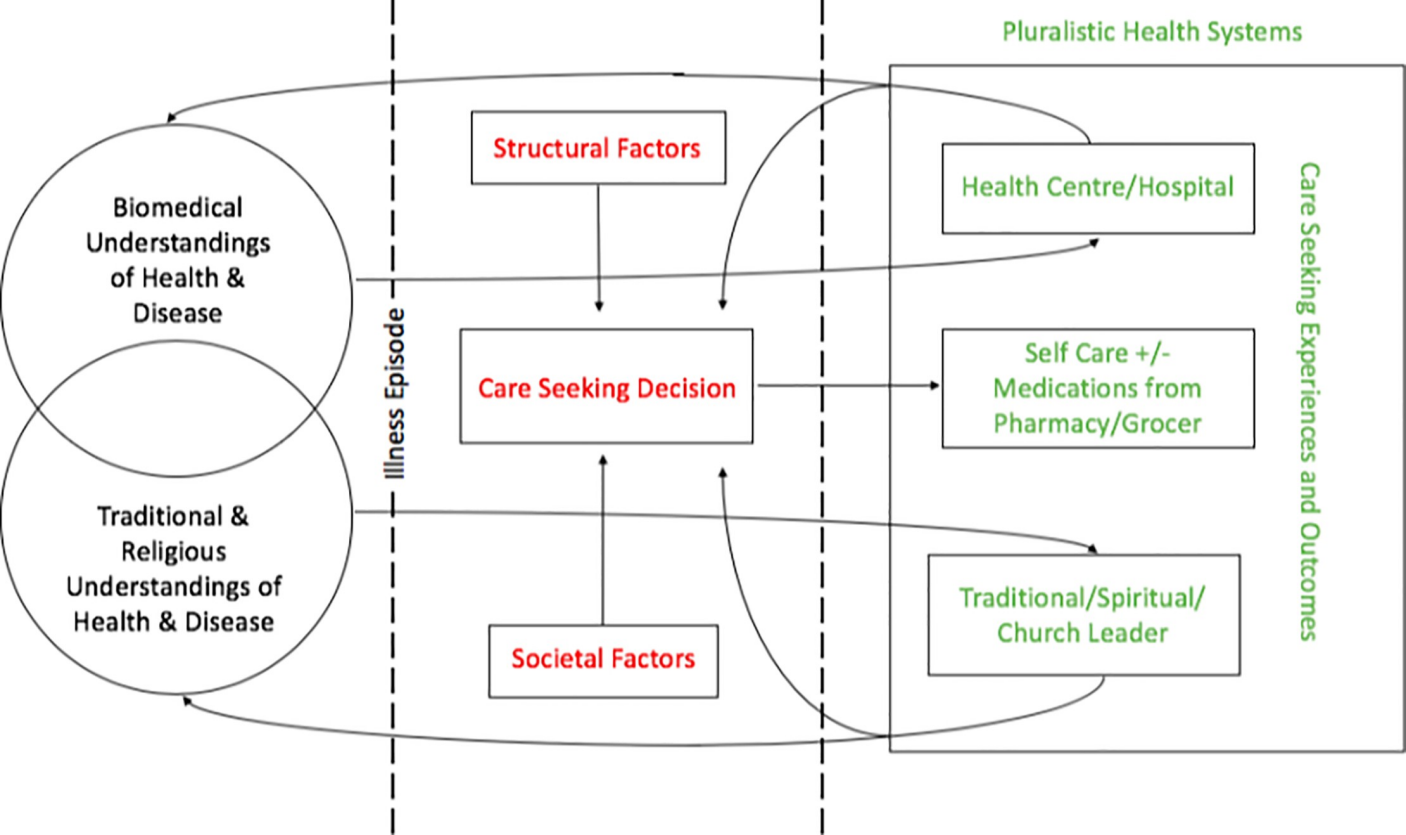
Proposed model of health seeking for chronic lung disease in Malawi.

Using the findings from our study, and building on existing determinant and systems models [15–18], in particular, the PASS model by Hausmann-Muela et al. [17], we present an adapted model to describe health seeking for chronic lung diseases in Malawi (Fig 1), highlighting how these processes are culturally, socially, and environmentally situated, and iteratively influenced by repeated episodes of help seeking.

This adapted model involves 3 sections: first, the underlying systems of understanding relating to health and disease, and second, triggered by an episode of illness in an individual, the factors influencing decisions around care-seeking. These include perceptions of the illness as well as societal and structural factors. Lastly, we consider the care-seeking episode in terms of the experience itself and its outcomes, and how these influence individual and societal illness understandings and, in turn, future care-seeking decisions. The influence of experience is particularly relevant to chronic lung disease trajectories, due to the likelihood of recurrent acute episodes of ill health.

#### Understandings of health and disease and their effects on health seeking

Our model acknowledges the significance of the various underlying systems of understanding relating to health and illness that exist in a sub-Saharan African setting such as Malawi–termed syncretic health beliefs. Syncretism has been described as “unifying or reconciling different or opposing schools of thought” [19]. Medical syncretism, first described by Muela et al. [20], here reflects the tendency for people to concurrently hold more than one system of understanding around health and illness. These systems inform illness interpretations and therefore shape health-seeking decisions made in a landscape characterised by medical pluralism. Describing the way in which individual illness perceptions stem from larger pre-existing systems of understanding, similar to Good’s ethnomedical system [18], our model depicts these various illness interpretations as relating to medical pluralism, making them pertinent throughout the experience of illness and health-seeking, not only as a source of service options. In a development from previous models, however, we show how individual health-seeking experiences and outcomes impact on these life-worlds [21], which are then reconstructed through such experiences.

Syncretic health beliefs were a key feature of participants’ accounts of illness in our research. Interactions between differing beliefs often took one of the following three forms: ‘division of labour’, parallel understandings of health and illness, and hierarchy of resort [22]. First, in a form of ‘division of labour’, different sources of help are sought depending on perceptions about the perceived illness-type. Traditional healers are consulted for symptoms understood to be underpinned by cultural concepts such as ‘mdulo’ [12, 13, 23], while health clinics are visited for illnesses expected to have biomedical explanations such as TB.

This system, in practice, means that biomedical knowledge levels amongst communities are important: where the relevant biomedical diagnosis is not commonly known (such as in the case of COPD in our study), biomedical care is often not considered. This also relates to the nature of the symptoms: participants in our study were less likely to consider biomedical explanations when initial symptoms were not felt to be severe or acute (such as for chronic cough). Improved awareness and understanding of chronic lung diseases amongst community members may, therefore, trigger earlier help seeking from formal health facilities for people with these symptoms, leading to improved disease management. This is, however, dependent on concurrent improvement in systems of diagnosis and management of these diseases, as well as improvements in accessibility, availability and acceptability of healthcare provision as discussed below.

Another form of syncretism involves people simultaneously drawing on different systems of understanding in attending to one illness. This is often seen where people use prayer/religious healing or visit traditional healers to address the perceived source of the disease (bewitching for instance), whilst attending a health centre or hospital for what they describe as the resulting illness, such as asthma. In these situations, traditional and religious beliefs do not present as barriers to accessing biomedical care and are likely to remain in place, even with increased presentation at formal health services. Given how traditional and religious healers are rooted in local communities, these existing structures may usefully be employed in efforts to deliver accessible healthcare for chronic lung diseases. This emphasises the importance of recognising and engaging with pluralistic systems where they exist in chronic lung disease management.

A final model of syncretism, which again arose in our data, is that of the hierarchy of resort [22].This phenomenon, most commonly seen in cases where participants, having tested negative for a given biomedical diagnosis (most often TB), sought further help from an alternative source such as a traditional healer, is well documented in the literature in a range of settings [24, 25]. A variant of such diagnosis seeking, is the ‘search for a cure’ from traditional healers, in response to a biomedical diagnosis of a chronic (non-curable) disease such as asthma. This too has been described in both Western and non-Western contexts [26–28]. In the context of the often insurmountable barriers to formal healthcare described in our data, these attempts to evade a lifelong sentence of recurrent illness episodes are particularly acute. Whilst education of health centre staff about chronic lung diseases and their management is again important here.

#### Societal factors: Social networks and their roles in health seeking

Authors of the PASS-model [17], building on Janzen’s concept of a Therapy Management Group [29], describe various ways in which significant others in communities contribute to and influence individuals’ health seeking decisions. These include advice and material support within social networks, social pressure to maintain normative behaviour, and stigma around socially discrediting illnesses. The specific expression of these influences varies between socio-cultural, economic and health systems contexts.

Our study revealed how, in line with many studies in LMIC settings, relatives and friends provided ‘therapy management’, encompassing both social support and contribution to decision-making around health seeking. In this specific context, there appeared to be a generational divide in advice given: family, namely parents and grandparents, were most often cited as reinforcing traditional understandings of health and disease encouraging health seeking from sources such as traditional healers, while friends tended to pass on biomedical systems of understanding to each other advocating attendance at formal health facilities. Feedback around health seeking experiences of societal groups, both positive and negative, was also commonly cited by participants as contributing to their own decision-making in times of illness.

These social features of the health seeking pathway may contribute to opportunities for strengthening local healthcare access and provision. This could firstly be achieved through improved routes of communication between the health system and a range of community members, including older ‘gatekeepers’ within therapy management groups. Second, sharing amongst social networks of positive experiences in local services for chronic illness diagnosis and management could then contribute to further integration of new services into the wider health system.

#### Structural factors affecting health seeking and the additional challenges in the case of chronic disease

Structural barriers to formal healthcare, incorporating the combined barriers posed by poor infrastructure (health service and transport related) and individual, household and community poverty, were central to participants’ accounts of accessing healthcare in our study. The often-cited example of having to borrow money to use a bicycle to access the hospital or health centre demonstrates how these factors are interlinked. These findings align with the existing literature on health seeking in Malawi, which cite factors including physical access and out of pocket expenditure as barriers to accessing healthcare in this context [30–32]: structural barriers which often feature in models of health seeking in sub-Saharan Africa [16]. Our data revealed how such barriers to physical accessibility intersected with previously-discussed factors relating to illness understandings, and negative experiences of stretched, understaffed services on arrival. This resulted in participants accessing alternative sources of help such as traditional healers, in the first instance.

In the case of chronic lung diseases such as asthma, characterised by multiple episodes of illness often requiring medication, these barriers were multiplied. Ongoing, repeated illness exacerbations in the context of structural barriers made it harder for participants to accept a diagnosis of chronic lung disease, accentuating their search for a curable diagnosis, as described above. Efforts to improve health systems in these settings should therefore aim primarily to address these initial barriers to care. Community management, through existing community health structures, could be an option for the management of chronic lung disease in Malawi to minimise the need to return to formal health facilities with every acute illness episode.

#### Care seeking experiences and their impacts on future episodes: Closing the loop

Experiences of self-care, or care seeking, from any source, and the outcome of these episodes, impact heavily on future care seeking decisions. This is important both in terms of the individual and through encounters involving their family and friends, either when accompanying patients to a consultation, or following sharing of health seeking experiences (not always linked to chronic lung disease) in societal groups. As in our study, these factors are often implicitly raised in the literature on health seeking, with reported shortages of equipment, staff and drugs at health centres leading to long waits, patients being turned away without care, and poor treatment by staff at health facilities [33, 34]. The impacts on subsequent health-seeking decisions are, however, rarely considered in existing models of health seeking, although they are reflected in the literature more generally [35].

Our model builds on existing models to reflect the continuity of people’s lived experiences of health and disease beyond the care seeking event, integrating feedback loops to illustrate the impacts of both positive and negative experiences on future health-seeking decisions. These may include factors relating to waiting times at facilities, shortages of staff, equipment or medication, and the perceived reception from practitioners, which may encourage or deter people from similar care seeking decisions in the future. These factors work synergistically with previously discussed structural barriers to health seeking, potentially compounding negative experiences and their deterrent effects.

Experiences and perceptions of ‘successful’ treatment, in terms of symptom resolution or improvement and communication around diagnoses are also important influences on both future care seeking and interpretation of illness. Resolution of symptoms following treatment by a traditional healer is likely to reinforce ethnomedical understandings, whilst a clearly explained diagnosis of asthma accompanied by a clear, effective management plan including accessible and affordable inhalers is likely to encourage the interpretation of symptoms and care-seeking within a biomedical model. For biomedical treatment to be experienced as ‘successful’ both structural access barriers and effective communication around diagnosis and management need to be addressed. This may require providers to pay more attention to psychosocial dimensions of illness within a context of pluralism and syncretic health beliefs.

### Study limitations

Despite attempts to include people with a range of disease symptoms and diagnoses within this study, some gaps in accessing participants remained. Firstly, we were only able to include patients who had accessed the health facility. This may mean that more marginalised people with greater barriers to access or utilisation of formal health facilities were overlooked. The use of FGDs with lay community members, as well as engagement with informal health providers, aimed to mitigate this limitation. The numbers of in-depth interviews carried out in this study were smaller than might be expected, particularly compared with research taking a more quantitative approach. In participant recruitment, purposive sampling was used to widen the scope of participant characteristics included in the study, and interviews and focus groups were continued to the point of data saturation, where no new findings were found to be emerging. This concept, derived from the grounded theory method [36], is customary across much of published qualitative research to date [37].

We were unable to confirm any illness diagnoses made, and as such had to rely on participant health passports. Given the weak and often insufficient diagnostic systems in place for chronic lung disease in this context, some diagnoses may be inaccurate. Nevertheless, it was clear that each of the participants faced significant challenges in relation to lung health and we feel this paper contributes to the improved documentation of their experiences. There may have also been some biases in the way sampling was derived for lay community member FGDs, due to our reliance on information provided by community leaders to assist in identification of participants. All the accounts of health seeking in this study were based on participants’ recall. Given the chronicity of their symptoms or conditions, we feel this does not present a significant challenge to the study, however, there may have been some merging of participant accounts in relation to specific illness episodes. We have aimed to provide detailed description of context throughout this manuscript to allow for the theoretical generalisability of this model to other low and middle-income settings.

## Conclusions and recommendations for action

Our study findings have shown how care seeking behaviour for chronic respiratory symptoms in Kasungu, Malawi is influenced by interactions between syncretic understandings of health and disease, social networks, including therapy management groups, and structural accessibility of different healthcare options within a pluralistic health system. As expressed in our adaptation of the PASS model [17], rather than separately shaping care seeking for individual illness episodes, individual and collective experiences of healthcare use further contribute to shaping future interpretations of symptoms and health seeking behaviour.

Efforts to improve effective and accessible healthcare provision for chronic lung disease need to address all the influences on health seeking behaviour identified. Improving the structural accessibility of formal health services is critical, including both geographic and financial accessibility. Expanding the remit of community health workers to facilitate two-way linkages between communities and the formal health system may contribute to addressing this, but only if sufficiently resourced and supported by the formal health system [38]. Engagement with other existing informal providers such as traditional healers may also help in the strengthening linkages to care throughout rural areas.

Improving communication between formal health providers, patients and wider communities around symptoms, diagnosis and management of chronic disease is required to support both timely care seeking and retention within the formal health system. Such communication is likely to be more effective if it is cognisant and respectful of both syncretic models of health and disease and pluralistic health seeking, rather than disrespectful or punitive with regard to traditional understandings. Community health workers may have an important role to play here with appropriate training.

Finally, the importance of experiences of care and perceived outcomes of treatment in shaping future care seeking for chronic disease underlines the necessity to improve not only the accessibility but the quality of diagnostic and management services, which will require further capacity building and training of formal healthcare providers, equipment and drug availability and attention to building the ‘software’ of health systems such as respectful and trusting relationships between providers and patients.

## Supporting information

S1 Appendix Research toolkit. Topic guides for focus group discussions and interviews.
(DOC)

S1 Table Supplementary quotes. (DOC)

## References

[1] Naghavi M, Forouzanfar MH. Burden of non-communicable diseases in sub-Saharan Africa in 1990 and 2010: Global Burden of Diseases, Injuries, and Risk Factors Study 2010. The Lancet. 2013;381:S95 10.1016/S0140-6736(13)61349-5

[2] World Bank. The Global Burden of Disease: Main Findings for Sub-Saharan Africa Washington, United States: The World Bank Group; 2013 [cited 2017 28th November 2017]. Available from: http://www.worldbank.org/en/region/afr/publication/global-burden-of-disease-findings-for-sub-saharan-africa.

[3] WHO. Asthma Fact sheet: World Health Organization; 2017 [cited 2017 19th December]. Available from: http://www.who.int/mediacentre/factsheets/fs307/en/.

[4] WHO. Chronic obstructive pulmonary disease (COPD) Fact sheet: World Health Organization; 2017 [cited 2017 19th December]. Available from: http://www.who.int/mediacentre/factsheets/fs315/en/.

[5] Mortimer K, Azhar H, Patel J, Kapur S, Gnatiuc L, Burney P, et al The burden of non-communicable lung disease in urban Malawi. European Respiratory Journal. 2015;46(suppl 59). 10.1183/13993003.congress-2015.PA1127

[6] Allain TJ, Aston S, Mapurisa G, Ganiza TN, Banda NP, Sakala S, et al Age Related Patterns of Disease and Mortality in Hospitalised Adults in Malawi. PLOS ONE. 2017;12(1):e0168368 10.1371/journal.pone.0168368 28099438 10.1371/journal.pone.0168368 PMC5242517

[7] Ahmed R, Robinson R, Mortimer K. The epidemiology of noncommunicable respiratory disease in sub-Saharan Africa, the Middle East, and North Africa. Malawi Medical Journal. 2017;29(2):203–11. PMC5610297. 28955434 10.4314/mmj.v29i2.24 PMC5610297

[8] Banda HT, Thomson R, Mortimer K, Bello GAF, Mbera GB, Malmborg R, et al Community prevalence of chronic respiratory symptoms in rural Malawi: Implications for policy. PLoS One. 2017;12(12):e0188437 Epub 2017/12/08. 10.1371/journal.pone.0188437 29216193 10.1371/journal.pone.0188437 PMC5720679

[9] National Statistical Office/Malawi, ICF. Malawi Demographic and Health Survey 2015–16 Zomba, Malawi: National Statistical Office and ICF, 2017.

[10] Malawi National Tuberculosis Control Programme Manual. Lilongwe, Malawi: Ministry of Health; 2012.

[11] Ritchie J, Spencer L, O' Connor W. "Carrying Out Qualitative Analysis" IN: Ritchie, J. and Lewis, J.—Qualitative research practice: a guide for social science students and researchers, pp. 219–262. In: Ritchie J, Lewis J editors. Qualitative research practice: a guide for social science students and researchers. London: SAGE; 2003.

[12] De Gabriele J. When Pills Don't Work: African Illnesses, Misfortune, and Mdulo. Religion in Malawi. 1999;(9):9–23.

[13] Steinforth AS. Troubled Minds: On the Cultural Construction of Mental Disorder and Normality in Southern Malawi: P. Lang; 2009.

[14] Morris B. CHEWA CONCEPTIONS OF DISEASE—SYMPTOMS AND ETIOLOGIES. The Society of Malawi Journal. 1985;38(1):14–36. 11618133

[15] Anderson RM. Revisiting the Behavioral Model and Access to Medical Care: Does it Matter? Journal of Health and Social Behavior. 1995;36(1):1–10. 10.2307/2137284 7738325

[16] Tomison T. Worlds Apart? Health-seeking behaviour and strategic healthcare planning in Sierra Leone. London: Development Studies Institute, London School of Economics and Political Science, 2013 January 2013. Report No.: Contract No.: No.13-139.

[17] Hausmann Muela S, Muela Ribera J, Toomer E, Peeters Grietens K. The PASS-model: a model for guiding health-seeking behavior and access to care research. Malaria Reports. 2012;2:e3(1):17–23.

[18] Good CM. Ethnomedical Systems in Africa: Patterns of Traditional Medicine in Rural and Urban Kenya: Guilford Publications; 1987.

[19] Pool R, Geissler W. Medical Anthropology: McGraw-Hill Education; 2005.

[20] Hausmann Muela S, Muela Ribera J, Mushi AK, Tanner M. Medical syncretism with reference to malaria in a Tanzanian community. Social Science & Medicine. 2002;55(3):403–13. 10.1016/S0277-9536(01)00179-4. 12144148

[21] Husserl E. The Crisis of European Sciences and Transcendental Phenomenology: An Introduction to Phenomenological Philosophy: Northwestern University Press; 1970.

[22] Romanucci-Ross L. Hierarchy Of Resort In Curative Practices: The Admiralty Islands, Melanesia. Journal Of Health And Social Behavior. 1969;Vol. 10(no. 3):201–9. 4309231

[23] Morris B. Chewa Medical Botany: A Study of Herbalism in Southern Malawi: Lit Verlag; 1996.

[24] Ewing VL, Tolhurst R, Kapinda A, SanJoaquin M, Terlouw DJ, Richards E, et al Understanding Interpretations of and Responses to Childhood Fever in the Chikhwawa District of Malawi. PLOS ONE. 2015;10(6):e0125439 10.1371/journal.pone.0125439 26087147 10.1371/journal.pone.0125439 PMC4472932

[25] Rao D. Choice of medicine and hierarchy of resort to different health alternatives among Asian Indian migrants in a metropolitan city in the USA. Ethnicity & health. 2006;11(2):153–67. Epub 2006/04/06. 10.1080/13557850500460306 . 16595317 10.1080/13557850500460306

[26] Mishel MH. Reconceptualization of the uncertainty in illness theory. Image J Nurs Sch. 1990;22(4):256–62. Epub 1990/01/01. . 2292449 10.1111/j.1547-5069.1990.tb00225.x

[27] Chen LC, Kleinman A, Ware NC. Health and Social Change in International Perspective Boston, Massachusetts: Harvard University Press; 1994.

[28] Våga BB. The search for care and cure: Exploring health seeking behaviour in Mbulu District, Tanzania. Bergen, Norway: University of Bergen; 2002.

[29] Janzen JM, Arkinstall W. The Quest for Therapy in Lower Zaire: University of California Press; 1978.

[30] Makwakwa L, Sheu M-l, Chiang C-Y, Lin S-L, Chang PW. Patient and health system delays in the diagnosis and treatment of new and retreatment pulmonary tuberculosis cases in Malawi. BMC Infectious Diseases. 2014;14(1):132 10.1186/1471-2334-14-132 24606967 10.1186/1471-2334-14-132 PMC3976046

[31] Wang Q, Brenner S, Leppert G, Banda TH, Kalmus O, De Allegri M. Health seeking behaviour and the related household out-of-pocket expenditure for chronic non-communicable diseases in rural Malawi. Health Policy Plan. 2015;30(2):242–52. Epub 2014/02/25. 10.1093/heapol/czu004 . 24561879 10.1093/heapol/czu004

[32] Ewing VL, Lalloo DG, Phiri KS, Roca-Feltrer A, Mangham LJ, SanJoaquin MA. Seasonal and geographic differences in treatment-seeking and household cost of febrile illness among children in Malawi. Malar J. 2011;10:32 Epub 2011/02/10. 10.1186/1475-2875-10-32 21303538 10.1186/1475-2875-10-32 PMC3049750

[33] Ingstad B, Munthali AC, Braathen SH, Grut L. The evil circle of poverty: a qualitative study of malaria and disability. Malaria Journal. 2012;11:15–. 10.1186/1475-2875-11-15 PMC3295708. 22236358 10.1186/1475-2875-11-15 PMC3295708

[34] Abiiro GA, Mbera GB, De Allegri M. Gaps in universal health coverage in Malawi: A qualitative study in rural communities. BMC Health Services Research. 2014;14(1):234 10.1186/1472-6963-14-234 24884788 10.1186/1472-6963-14-234 PMC4051374

[35] Colvin CJ, Smith HJ, Swartz A, Ahs JW, de Heer J, Opiyo N, et al Understanding careseeking for child illness in sub-Saharan Africa: A systematic review and conceptual framework based on qualitative research of household recognition and response to child diarrhoea, pneumonia and malaria. Social Science & Medicine. 2013;86:66–78. 10.1016/j.socscimed.2013.02.031. 23608095

[36] Glaser B, Strauss A. The discovery of grounded theory: Strategies for qualitative research New York: Aldine Publishing Company; 1967.

[37] Saunders B, Sim J, Kingstone T, Baker S, Waterfield J, Bartlam B, et al Saturation in qualitative research: exploring its conceptualization and operationalization. Quality & quantity. 2018;52(4):1893–907. Epub 2017/09/14. 10.1007/s11135-017-0574-8 . 29937585 10.1007/s11135-017-0574-8 PMC5993836

[38] Theobald S, MacPherson E, McCollum R, Tolhurst R. Close to community health providers post 2015: Realising their role in responsive health systems and addressing gendered social determinants of health. BMC Proceedings. 2015;9(10):S8 10.1186/1753-6561-9-s10-s8 28281706 10.1186/1753-6561-9-S10-S8 PMC4699124

