# Supplementary material for: Health seeking for chronic lung disease in central Malawi: Adapting existing models using insights from a qualitative study
Source: PLoS One. 2018 Dec 17;13(12):e0208188. doi: 10.1371/journal.pone.0208188 (PMC6296555; doi:10.1371/journal.pone.0208188)
Supplement: S1 Appendix — Topic guides for focus group discussions and interviews. (DOC) [file pone.0208188.s001.doc]

# **S2 Appendix. Research Toolkit**

# **Contents**

[Focus Group Discussion Guide for Community Members 2](#__RefHeading___Toc530574799)

[Focus Group Discussion Guide for Informal Health Care Providers 11](#__RefHeading___Toc530574800)

[Focus Group Discussion Guide for Health Surveillance Assistants 21](#__RefHeading___Toc530574801)

[Focus Group Discussion Guide for Village Health Committee Members 31](#__RefHeading___Toc530574802)

[Interview Guide for Health Care Workers 40](#__RefHeading___Toc530574803)

[Key informant interview guide for Traditional Leaders 50](#__RefHeading___Toc530574804)

[In-depth Interview Guide for Patient with TB or CAD 57](#__RefHeading___Toc530574805)

## Focus Group Discussion Guide for Community Members

Prior to the focus group beginning I would just like to ask you a few demographic questions. ***(These questions should be asked of the participant prior to being in the main focus group as the questions are personal).***

Remember to give each participant a number that will act as an identity (ID) throughout the FGD.

### Demographics

**What is your educational level?**

**What do you do to earn an income?**

**Are you male or female?**

**How old are you?**

**What is your religion?**

**What is your tribe?**

### Introduction to the Focus Group

Thank you for taking the time to meet with me today. I hope you will think of this focus group as an opportunity to share your communities’ experiences. I hope that you feel able to express your opinions openly and honestly. Within the focus group I will ask you a series of questions around different topic areas. Firstly, I would like to ask you a few questions about your knowledge of chronic cough and breathlessness

### Knowledge of Lung Health

1. When would you call a cough a chronic one?

PROMPT:

- How long would someone have to have been coughing before you would say it was chronic?

**Kodi chifuwa mumachitcha chamgonagona pakapita nthawi yayitali bwanji?(Funsitsani za nthawi yomwe munthuyo wakhala akukhosomola)**

1. What diseases do you know are associated with chronic cough?

PROMPT:

- TB, Asthma, Other CAD?

**Kodi ndi matenda anji omwe amagwirizana ndi chifuwa cha mgonagona? Funsitsani za mphumu kapenanso befu (chifuwa chotha phuma) (wheezing)**

1. How would you describe breathlessness or wheezing?

**Kodi befu ndi chiyani?**

1. What diseases do you know that are associated with difficulty in breathing?

PROMPT:

- - TB, Asthma, other CAD?

**Kodi ndi matenda anji omwe amakhudzana ndi befu**

**Funsitsani za**

- **TB, Asthma ndi CAD**

1. Would you tell us what you know about XX? (*Instruction to researcher: XX refers to each of the diseases of interest that have been mentioned in question 2. You should ask about them in order: TB, Asthma,CAD.)*

PROMPT:

- What are the causes of these diseases?
- What are the signs and symptoms?
  - Is this disease associated with shortness of breath?
    - If yes, how do you tell if someone has shortness of breath?
- How are they transmitted?
- How are they prevented?
- How do you know if someone has this disease?
- What is the treatment for these diseases?

**Mungatiuzeko zomwe mukudziwa za XX (*Instruction to researcher: XX refers to each of the diseases of interest that have been mentioned in question 2. You should ask about them in order: TB, Asthma, CAD.)***

- - - **Kodi zomwe zimayambitsa matenda amenewa ndi chiyani?**
    - **Zizindikiro zake ndi zotani?**
      - **Kodi matenda amenewa amakhudzana ndi befu?**
      - **Mungadziwe bwanji kuti munthu ali ndi befu?**
    - **Kufala kwake**
    - **Kupewa kwake**
    - **Mungadziwe bwanji kuti munthu ali ndi matenda amenewa?**
    - **Mankhwala amatenda amenewa ndi chiyani?**

1. Where do you get the information you have shared with me about lung health?

**Kodi zimene mwafotokozazi zokhudzana ndi matenda omwe amagwira mapapo mumazimvera kuti?**

PROMPT:

- Within your community, are there groups that provide information about lung health?
  - Can you tell me who they are? Name them?

**Kodi mdera/mmudzi muno ndi magulu ati omwe amafalitsa uthenga wokhudzana ndi matenda omwe amagwira mapapo (ie TB chronic cough)?**

**Ndi ndani?**

- - 1. If so, how do they provide this information?
       1. Drama, song, leaflet, talk, poster, radio etc.

**Ngati alipo, amafalitsa motani( ieDrama, song, leaflet, talk, poster, radio)?**

- - 1. Which group gives a clearer understanding of lung health issues? Explain?

**Mwamagulu omwe mwatchulawa ndi magulu ati omwe amafalitsa mauthengawa momveka bwino kwambiri? Fotokozani?**

- - 1. Which method(s) gives a clearer understanding of lung health issues? Explain?

**Ndi gulu liti komanso ndi njira ziti zimene zimapereka uthengawu momveka bwino kwambiri pa nkhani ya matenda omwe amagwira mapapo? Fotokozani?**

1. What recommendations can you give to improve communication on health information focusing on lung health?

**Munganenepo zotani pofuna kupititsa mtsogolo kafalitsidwe ka mauthenga okhudzana ndi matenda omwe amagwira mapapo (TB, asthma komanso chifuwa cha mgonagona)**

PROMPT:

- How would you like to receive this information in the future?

**Mungakonde mutamaulandira bwanji uthenga umenewu mtsogolo muno?**

- Is there any specific areas that you would like additional information on?

**Pali zinthu zina zimene mungafune uthenga wina owonjezera?**

***Summarise key points from knowledge discussion.***

***Thank you very much for providing me with information on your knowledge of lung health, unless you want to add anything in this area, I would like to ask you some more questions about how people seek health care when they get sick in this community.***

### Pathways to Care Seeking

1. What do people in this community do when they get sick?

**Kodi kunoko anthu akadwala amatani?**

PROMPT:

- What type of care do they seek?
  - Self treatment, traditional methods, formal health care, faith healing

**Kodi amakafuna thandizo kuti? Funsitsani za kupeza thandizo kwa asing’anga, ku chipatala, mapemphero komanso kuzithandiza okha pakhomo.**

- - Why this type of care?
- **Chifukwa chiyani amasankha kutero?**
- How do they decide to seek care?

**Kodi ndi ndani amapanga ganizo lopita kukafuna thandizolo?**

- - Who is involved in the decision?

**Ndi anthu ati omwe amakhudzidwa ndi kupanga chiganizocho?**

- - What factors are important to consider in the decision to seek care?
    - E.g. accessibility, distance, cost

**Ndi zinthu ziti zomwe anthu amaona akafuna kupanga chiganizo cha kokalandira thandizo la za aumoyo?**

**2.** If someone has a chronic cough in this community, what do they do?

PROMPT:

- Where do they go?
- Who do they speak to?
- When do they do this?
- Who is involved in this?
- How is this different to when people get sick with other diseases?

**Kodi mdera lino, munthu akakhala ndi chifuwa cha mgonagona amatani?**

- **Amapita kuti kukafuna thandizo la zaumoyo**
- **Amayamba kufuna thandizo pakapita nthawi yayitali bwanji?**
- **Amafunsa ndani popanga chiganizo choterechi?**
- **Amakhudzidwa ndi ndani popanga chiganizocho?**
- **Kodi zimasiyana akakhala matenda ena?**

**3.** If someone has difficulty breathing in this community, what do they do?

PROMPT:

- Where do they go?
- Who do they speak to?
- When do they do this?
- Who is involved in this?
- How is this different to when people get sick with other diseases?

**Nanga munthu akakhala ndi befu amatani?**

- **Amapita kuti?**
- **Amafunsakwandani?**
- **Amayamba kufunsa pakapita nthawi yayitali bwanji?**
- **Amafunsa ndani popanga chiganizo choterechi?**
- **Amakhudzidwa ndi ndani popanga chiganizocho?**
- **Kodi zimasiyana akakhala matenda ena?**

**4.** In general, what are the communities’ perceptions of formal health care?

- - 1. How does this vary between public and private providers?
    2. How does this vary based on the type of service?

**Kodi anthu kunoko chithandizo chomwe chimaperekedwa ku chipatala amachiona bwanji?**

- **Nanga pali kusiyana pakati pa zipatala za boma ndi zipatala zolipira?**
- **Nanga zimasiyana bwanji? (kutengera ndi mtundu wa thandizo lomwe amapereka?)**

**5.** What would you say are the main challenges faced by people in this community when seeking health care?

**Nanga ndi mavuto otani omwe anthu akuno amakumana nawo pokafuna thandizo kuchipatala?**

PROMPT:

- How does this differ between formal and informal health care?

**Pali kusiyana kotani pakati pa ku chipatala ndi thandizo lina ie kwa asing’anga**

- How does this differ between the public and private health facilities?

**Pali kusiyana pakati pa zipatala za boma ndi zipatala zolipira**

- Would you suggest solutions to various challenges people face to access health services?

**Mukuona ngati tingatani pothetsa mavutowa?**

6. Over the last 2 years, what changes have you seen in the choice of providers available in your community?

- What can you say about the quality of the services that are provided?

**Kodi mu zaka ziwiri zapitazi pali kusintha kwina kulikonse pa za komwe anthu amapita kukapezako thandizo la za umoyo?**

- **Munganeneko chiyani pa zakolite yathandizo lomwe limaperekedwa kumeneko?**

***Summarise key points from pathways to care seeking discussion.***

***Thank you very much for providing me with information on your knowledge of care seeking behaviour, unless you want to add anything in this area, I would like to ask you some more questions about informal health providers in your community.***

### Perceptions of Informal Health Care Providers (NB: HSAs are not IHPs)

1. What do you understand by the term informal health provider?

**Kodi mumamvetsa kuti mavolontiya a zaumoyo kumudzi ndi ndani?**

1. Can you tell us the different IHPs that are in this community? **(*Instruction to the researcher: ask the below in relation to each of the IHPs mentioned.)***

- What services do they offer?
  - Information (what diseases?), referrals etc.
  - Do people use these services? Why/why not?
- What additional services could they provide that they are not already?
- What could be done to improve the quality of the services they provide?

**Kodi ndi magulu ati a mavolontiya a zaumoyo omwe alipo kudera lino?**

- **Amapereka thandizo lanji?**
  - **Pa matenda anji? Amatha kutumiza odwala ku chipatala (referral)?**
  - **Nanga anthu amagwiritsa ntchito upangiriwo/malangizowo? Fotokozani?**
- **Kupatula ntchito zomwe akugwira mavololontiya a za umoyo akumudziwa, pali ntchito zina zomwe angagwirenso zokhudzana ndi za umoyo? Fotokozani?**
- **Pachitike chiyani kuti iwowa azipereka thandizo labwino (kolite)?**

1. Who are the target populations of these IHPs?
   - Are there any groups in your community or neighbouring communities left out of access to IHP services?
   - If so which are these groups?
   - Explain?

**Kodi ntchito zawo amafikira nazo ndani?**

- **Kodi alipo magulu ena a anthu mdera lino kapena madera oyandikana nawo omwe safikiridwa ndi thandizo loperekedwa ndi mavolontiya amenewa?**
- **Ngatialipo, ndiati? Chifukwa chiyani? Fotokozani.**

1. What is your perception of IHPs and the services they offer?

**Kodi thandizo lomwe mavolontiya a zaumoyo amm’idzi amapereka lino mumaliwona bwanji?**

- - Are there cases in your community where IHPs’ services are shunned? Explain? – Prompt on the status of the provider i.e HIV, economic status, education levels, tribe etc
- **Kodi zimachitika kuti thandizo lomwe mavolontiya a za umoyo amapereka limakanidwa? Fotokozani.**

**Funsitsani potengera:**

- **maonekedwe,**
- **maphunziro**
- **kupata kwa munthu,**
- **kasunga chinsinsi**
- **mtundu wa munthu**
- **kolite ya chithandizo chomwe amapereka**

***Summarise key points from IHP discussion.***

***Thank you very much for providing me with information on your knowledge of IHPs, unless you want to add anything in this area.***

## Focus Group Discussion Guide for Informal Health Care Providers

Prior to the focus group beginning I would just like to ask you a few demographic questions. ***(These questions should be asked of the participant prior to being in the main focus group as the questions are personal)***

### Demographics

**What is your educational level?**

**Do you work?**

**If yes, what is your occupation?**

**Are you male or female?**

**How old are you?**

**What is your religion?**

**What is your tribe?**

**What type of informal health care do you provide?**

**How long have you been an informal health care provider?**

**Have you ever received any training to be an informal health care provider?**

### Introduction to the Focus Group

Thank you for taking the time to meet with me today. I hope you will think of this focus group as an opportunity to share your communities’ experiences and your experience of being an IHP. I hope that you feel able to express your opinions openly and honestly. Within the focus group I will ask you a series of questions based around different topic areas. Firstly, I would like to ask you a few questions about your knowledge of chronic cough and breathlessness.

### Knowledge of Lung Health

**1.** When would you call a cough a chronic one?

PROMPT:

- How long would someone have to have been coughing before you would say it was chronic?

**Kodi chifuwa mumachitcha chamgonagona pakapita nthawi yayitali bwanji?(Funsitsani za nthawi yomwe munthuyo wakhala akukhosomola)**

**2.** What diseases do you know are associated with chronic cough?

PROMPT:

- TB, Asthma, other CAD?

**Kodi ndi matenda anji omwe amagwirizana ndi chifuwa cha mgonagona? Funsitsani za mphumu kapenanso befu (chifuwa chotha phuma) (wheezing)**

1. How would you describe breathlessness or wheezing?

**Kodi befu ndi chiyani?**

1. What diseases do you know that are associated with difficulty in breathing?

PROMPT:

- TB, Asthma, other CAD?

**Kodi ndi matenda anji omwe amakhudzana ndi befu**

**Funsitsani za**

- **TB, Asthma ndi CAD**

5. Would you tell us what you know about X? (*Instruction to researcher: X refers to each of the diseases of interest that have been mentioned in question 2. You should ask about them in order: TB, Asthma, other CAD)*

PROMPT:

- What are the causes of these diseases?
- What are the signs and symptoms?
  - Is this disease associated with shortness of breath?
    - If yes, how do you tell if someone has shortness of breath?
- How are they transmitted?
- How are they prevented?
- How do you know if someone has this disease?
- What is the treatment for these diseases?

**Mungatiuzeko zomwe mukudziwa za XX (*Instruction to researcher: XX refers to each of the diseases of interest that have been mentioned in question 2. You should ask about them in order: TB, Asthma, CAD.)***

- - - **Kodi ndi chiyani chomwe chimayambitsa matenda amenewa?**
    - **Zizindikiro zake ndi zotani?**
      - **Kodi matenda amenewa amakhudzana ndi befu?**
      - **Mungadziwe bwanji kuti munthu ali ndi befu?**
    - **Kufala kwake**
    - **Kupewa kwake**
    - **Mungadziwe bwanji kuti munthu ali ndi matenda amenewa?**
    - **Mankhwala amatenda amenewa ndi chiyani?**

6. Where did you get the information you have shared with me about lung health?

**Kodi zimene mwafotokozazi zokhudzana ndi matenda omwe amagwira mapapo mumazimvera kuti?**

PROMPT:

- Within your community, are there groups that provide information about lung health?
  - Can you tell me who they are? Name them?

**Kodi mdera/mmudzi muno ndi magulu ati omwe amafalitsa uthenga wokhudzana ndi matenda omwe amagwira mapapo (ie TB chronic cough)?**

**Ndi ndani?**

- - 1. If so, how do they provide this information?
       1. Drama, song, leaflet, talk, poster, radio etc.

**Ngati alipo, amafalitsa motani( ieDrama, song, leaflet, talk, poster, radio)?**

- - 1. Which group gives a clearer understanding of lung health issues? Explain?

**Mwamagulu omwe mwatchulawa ndi magulu ati omwe amafalitsa mauthengawa momveka bwino kwambiri? Fotokozani?**

- - 1. Which method(s) gives a clearer understanding of lung health issues? Explain?

**Ndi gulu liti komanso ndi njira ziti zimene zimapereka uthengawu momveka bwino kwambiri pa nkhani ya matenda omwe amagwira mapapo? Fotokozani?**

1. What recommendations can you give to improve communication on health information focusing on lung health?

**Munganenepo zotani pofuna kupititsa mtsogolo kafalitsidwe ka mauthenga okhudzana ndi matenda omwe amagwira mapapo (TB, asthma komanso chifuwa cha mgonagona)**

PROMPT:

- How would you like to receive this information in the future?

**Mungakonde mutamaulandira bwanji uthenga umenewu mtsogolo muno?**

- Is there any specific areas that you would like additional information on?

**Pali zinthu zina zimene mungafune uthenga wina owonjezera?**

***Summarise key points from knowledge discussion.***

***Thank you very much for providing me with information on your knowledge of lung health, unless you want to add anything in this area, I would like to ask you some more questions about how people seek health care when they get sick in this community.***

### Pathways to Care Seeking

1. What do people in this community do when they get sick?

**Kodi kunoko anthu akadwala amatani?**

PROMPT:

- What type of care do they seek?
  - Self treatment, traditional methods, formal health care, faith healing

**Kodi amakafuna thandizo kuti? Funsitsani za kupeza thandizo kwa asing’anga, ku chipatala, mapemphero komanso kuzithandiza okha pakhomo.**

- - Why this type of care?

**Chifukwa chiyani amasankha kutero?**

- How do they decide to seek care?

**Kodi ndi ndani amapanga ganizo lopita kukafuna thandizolo?**

- - Who is involved in the decision?

**Ndi anthu ati omwe amakhudzidwa ndi kupanga chiganizocho?**

- - What factors are important to consider in the decision to seek care?
    - E.g. accessibility, distance, cost

**Ndi zinthu ziti zomwe anthu amaona akafuna kupanga chiganizo cha kokalandira thandizo la za aumoyo?**

2. If someone has a chronic cough in this community, what do they do?

PROMPT:

- Where do they go?
- Who do they speak to?
- When do they do this?
- Who is involved in this?
- How is this different to when people get sick with other diseases?

**Kodi mdera lino, munthu akakhala ndi chifuwa cha mgonagona amatani?**

- **Amapita kuti kukafuna thandizo la zaumoyo**
- **Amayamba kufuna thandizo pakapita nthawi yayitali bwanji?**
- **Amafunsa ndani popanga chiganizo choterechi?**
- **Amakhudzidwa ndi ndani popanga chiganizocho?**
- **Kodi zimasiyana akakhala matenda ena?**

3. If someone has difficulty breathing in this community, what do they do?

PROMPT:

- Where do they go?
- Who do they speak to?
- When do they do this?
- Who is involved in this?
- How is this different to when people get sick with other diseases?

**Nanga munthu akakhala ndi befu amatani?**

- **Amapita kuti?**
- **Amafunsakwandani?**
- **Amayamba kufunsa pakapita nthawi yayitali bwanji?**
- **Amafunsa ndani popanga chiganizo choterechi?**
- **Amakhudzidwa ndi ndani popanga chiganizocho?**
- **Kodi zimasiyana akakhala matenda ena?**

1. In general, what are this communities’ perceptions of formal health care?

**Kodi anthu kunoko chithandizo chomwe chimaperekedwa ku chipatala amachiona bwanji?**

- - 1. How does this vary between public and private providers?

**Nanga pali kusiyana pakati pa zipatala za boma ndi zipatala zolipira?**

- - 1. How does this vary based on the type of service?

**Nanga zimasiyana bwanji? (kutengera ndi mtundu wa thandizo lomwe amapereka?)**

1. Over the last 2 years, what changes have you seen in the choice of providers available in your community?

**Kodi mu zaka ziwiri zapitazi pali kusintha kwina kulikonse pa za komwe anthu amapita kukapezako thandizo la za umoyo?**

- What can you say about the quality of the services that are provided?

**Munganeneko chiyani pa zakolite yathandizo lomwe limaperekedwa kumeneko?**

6. What would you say are the main challenges faced by people in this community when seeking health care?

**Nanga ndi mavuto otani omwe anthu akuno amakumana nawo pokafuna thandizo kuchipatala?**

PROMPT:

- How does this differ between formal and informal health care?

**Pali kusiyana kotani pakati pa ku chipatala ndi thandizo lina ie kwa asing’anga**

- How does this differ between the public and private health facilities?

**Pali kusiyana pakati pa zipatala za boma ndi zipatala zolipira**

- Would you suggest solutions to various challenges people face to access health services?

**Mukuona ngati tingatani pothetsa mavutowa?**

***Summarise key points from pathways to care seeking discussion.***

***Thank you very much for providing me with information on your knowledge of care seeking behaviour, unless you want to add anything in this area, I would like to ask you some more questions about how people seek health care when they get sick in the communities where you work informal health providers in the communities where you work.***

### Perceptions of Informal Health Care Providers (NB: HSAs are not IHPs)

1. What do you understand by the term informal health provider?

**Kodi mumamvetsa kuti mavolontiya a zaumoyo kumudzi ndi ndani?**

1. Can you tell us the different IHPs that are in this community? (*Instruction to the researcher: ask the below in relation to each of the IHPs mentioned.)*

**Kodindimaguluatiothandizirantchitozaumoyoomwealipokudera lino?**

- What services do they offer?
  - Information (what diseases?), referrals etc.
  - Do people use these services? Why/why not?
- What additional services could they provide that they are not already?
- What could be done to improve the quality of the services they provide?

**Kodi ndi magulu ati a mavolontiya a zaumoyo omwe alipo kudera lino?**

- **Amapereka thandizo lanji?**
  - **Pa matenda anji? Amatha kutumiza odwala ku chipatala (referral)?**
  - **Nanga anthu amagwiritsa ntchito upangiriwo/malangizowo? Fotokozani?**
- **Kupatula ntchito zomwe akugwira mavololontiya a za umoyo akumudziwa, pali ntchito zina zomwe angagwirenso zokhudzana ndi za umoyo? Fotokozani?**
- **Pachitike chiyani kuti iwowa azipereka thandizo labwino (kolite)?**

1. Who are the target populations of these IHP?
   - Are there any groups in your community or neighbouring communities left out of access to IHP services?
   - If so which are these groups?
   - Explain?

**Kodi ntchito zawo amafikira nazo ndani?**

- **Kodi alipo magulu ena a anthu mdera lino kapena madera oyandikana nawo omwe safikiridwa ndi thandizo loperekedwa ndi mavolontiya amenewa?**
- **Ngatialipo, ndiati? Chifukwa chiyani? Fotokozani.**

1. Do you work with formal health care providers?

**Kodi mumagwira ntchito ndi azachipatala? Mumagwira nawo bwanji?**

- If yes, in what ways do you work with them? Explain

**Mumagwira nawo bwanji?**

- What are the advantages of working with formal health care providers?

**Pali ubwino wanji wogwira ndi achipatala amenewa?**

- What are the challenges of working with formal health care providers?

**Nanga kuipa kwake ndi kotani? Mavuto ogwira ntchito ndi achipatala?**

- - - How could these challenges be overcome?

**Mavuto amenewa angathetsedwe bwanji?**

- If no, in the future how do you think IHPs could work with the formal health care system?

**Ngati sagwira ntchito ndi a chipatala mukuganiza kuti mtsogolo muno mungadzagwire nawo bwanji?**

1. In your opinion, how do community members perceive the services offered by IHPs?

**Kodi maganizo anu ndi otani pa za ntchito zimene mavolontiya amagwira?**

- - What support do you receive from the community to fulfil your role?

**Ngati mavolontiya anthu amakuthandizirani motani kuti mukwaniritse bwino ntchito zanu?**

- - Are there cases in your community where IHPs’ services are shunned? Explain? – Prompt on the status of the provider i.e HIV, economic status, education levels etc

**Kodi zimachitika kuti thandizo lomwe mavolontiya a za umoyo amapereka limakanidwa? Fotokozani.**

**Funsitsani potengera:**

- **maonekedwe,**
- **maphunziro**
- **kupatakwamunthu,**
- **kasungachinsinsi**
- **mtundu wa munthu**
- **kolite ya chithandizo chomwe amapereka**

1. In general, what training do informal health care providers receive?

**Kodi mavolontiya amalandira upangiri / maphunziro/ (training) wotani?**

- - Who from?

**Amawaphunzitsa ndi ndani?**

- - What additional training do you think would be necessary?

**Ndi maphunzironso ati amene mukuona kuti ndi ofunika kwambiri?**

- - 1. Specific health areas?

**Makamaka mbali iti ya za umoyo?**

1. What incentive do you receive to be an informal health provider?

**Pali chilichonse chimene mumalandirako/mumapezako ngati volontiya?**

- - Financial/non-financial?

**Ndalama/ non monetary**

1. What challenges do you face when fulfilling your role as an informal health provider?

**gati volontiya nNdi mavuto anji omwe mumakumana nawo pogwira ntchito?**

***Summarise key points from IHP discussion.***

***Thank you very much for providing me with information on your knowledge of IHPs, unless you want to add anything in this area.***

***Is there anything you would like to add regarding any of the topics we have discussed? Many thanks for your time today it has been most useful to our study.***

## Focus Group Discussion Guide for Health Surveillance Assistants

Prior to the focus group beginning I would just like to ask you a few demographic questions. ***(These questions should be asked of the participant prior to being in the main focus group as the questions are personal)***

### Demographics

**What is your educational level?**

**What are your main roles and responsibilities?**

**What is the name of the catchment area where you work?**

**Are you male or female?**

**How old are you?**

**For how long have you been working as an HSA?**

### Introduction to the Focus Group

Thank you for taking the time to meet with me today. I hope you will think of this focus group as an opportunity to share your experiences as a health surveillance assistant. I hope that you feel able to express your opinions openly and honestly. Within the focus group I will ask you a series of questions based around different topic areas. Firstly, I would like to ask you a few questions about your knowledge of Chronic Cough and breathlessness.

### Knowledge of Lung Health

1. When would you call a cough a chronic one?

PROMPT:

- How long would someone have to have been coughing before you would say it was chronic?

**Kodi chifuwa mumachitcha cha mgonagona patapita nthawi yayitali bwanji? (funsitsani za nthawi yomwe munthuyo amakhala akukhosomola)**

1. What diseases do you know are associated with chronic cough?

PROMPT:

- TB, Asthma, other CAD?

**Kodi ndi matenda anji omwe amagwirizana ndi chifuwa chamgonagona? Funsitsani za mphumu kapenanso befu (chifuwa chotha phuma)**

1. How would you describe breathlessness or wheezing?

**Kodi befu ndi chiyani?**

1. What diseases do you know that are associated with difficulty in breathing?

PROMPT:

- TB, Asthma, other CAD?

**Kodi ndi matenda anji omwe amakhudzana ndi befu**

**Funsitsani za**

- **TB, Asthma, other CAD**

1. Would you tell us what you know about X? (*Instruction to researcher: X refers to each of the diseases of interest that have been mentioned in question 2 and 4. You should ask about them in order: TB, Asthma, Other CAD?)*

PROMPT:

- What are the causes of these diseases?
- What are the signs and symptoms?
  - Is this disease associated with shortness of breath?
    - If yes, how do you tell if someone has shortness of breath?
- How are they transmitted?
- How are they prevented?
- How do you know if someone has this disease?
- What is the treatment for these diseases?

**Mungatiuzeko zomwe mukudziwa za XX (*Instruction to researcher: XX refers to each of the diseases of interest that have been mentioned in question 2. You should ask about them in order: TB, Asthma, CAD.)***

- - - **Zomwe zimayambitsa matenda amenewa**
    - **Zizindikiro zake ndizotani?**
    - **Kufala kwake**
    - **Kupewa kwake**
    - **Mungadziwe bwanji kuti munthu ali ndi matenda amenewa?**
    - **Mankhwala amatenda amenewa ndi chiyani?**

1. Where did you get the information you have shared with me about lung health?

**Kodi zimene mwafotokozazi zokhudzana ndi matenda omwe amagwira mapapo mumazimvera kuti?**

PROMPT:

- Within the catchment area of this facility, are there groups that provide information about lung health?
  - Can you tell me who they are? Name them?

**Kodi mdera lozungulira chipatala chino muli magulu/anthu ati omwe amafalitsa mauthengawa? ( ie asthma, TB ndi Chronic cough)?**

**Ndi ndani?**

- - - If so, how do they provide this information?
      - Drama, song, leaflet, talk, poster, radio etc.

**Ngati alipo, amafalitsa motani?**

- - - Which group gives a clearer understanding of lung health issues? Explain?

**Mwamagulu mwatchulawa ndimagulu ati omwe amafalitsa mauthengawa momveka bwino kwambiri?**

**Fotokozani?**

- - - Which method(s) gives a clearer understanding of lung health issues? Explain?

**Ndi njira ziti zimene zimapereka uthengawu momveka bwino kwambiri?**

**Fotokozani?**

1. What recommendations can you give to improve communication on health information focusing on lung health?

**Munganenepo zotani pofuna kupititsa patsogolo kafalitsidwe ka mauthenga okhudzana ndi matenda omwe amagwira mapapo (TB, asthma komanso chifuwa cha mgonagona)**

PROMPT:

- How would you like to receive this information in the future?

**Pali zinthu zina zimene mungafune uthenga wina owonjezera?**

- Are there any specific areas that you would like additional information on?

**Kodi palinso Madera ena omwe mungafune uthenga oonjezera?**

***Summarise key points from knowledge discussion.***

***Thank you very much for providing me with information on your knowledge of lung health, unless you want to add anything in this area, I would like to ask you some more questions about how people seek health care when they get sick in the communities where you work.***

### Pathways to Care Seeking

1. What do people in this community do when they get sick?

**Kodi kunoko anthuakadwalaamatani?**

PROMPT:

- What type of care do they seek?

**Kodi amakafuna thandizo kuti?**

- - Self treatment, traditional methods, formal health care, faith healing

**Funsitsani za kupeza thandizo kwa asing’anga, ku chipatala, mapemphero komanso kuzithandiza okha pakhomo.**

- - Why this type of care?
- **Chifukwachiyaniamasankhakutero?**
- How do they decide to seek care?

**Kodi amapanga bwanji ganizo lofuna thandizo la zaumoyo?**

- - Who is involved in the decision?

**Ndi anthu ati omwe amakhudzidwa ndi kupanga chiganizocho?**

- - What factors are important to consider in the decision to seek care?
    - E.g. accessibility, distance, cost

**Ndi zinthu ziti zomwe anthu amaona akafuna kupanga chiganizo cha kokalandira thandizo la za umoyo?**

1. If someone has a chronic cough in this community, what do they do?

PROMPT:

- Where do they go?
- Who do they speak to?
- When do they do this?
- Who is involved in this?
- How is this the same as when people get sick with other things?
- How is this different to when people get sick with other things?

**Kodi mdera lino, munthu akakhala ndi chifuwa cha mgonagona amatani?**

- **Amapita kuti kukafuna thandizo la zaumoyo**
- **Amayamba kufuna thandizo pakapita nthawi yayitali bwanji?**
- **Amafunsa ndani popanga chiganizo choterechi?**
- **Amakhudzidwa ndi ndani popanga chiganizocho?**
- **Kodi zimasiyana akakhala matenda ena?**

1. If someone has difficulty breathing in this community, what do they do?

PROMPT:

- Where do they go?
- Who do they speak to?
- When do they do this?
- Who is involved in this?
- How is this different to when people get sick with other diseases?

**Nanga munthu akakhala ndi befu amatani?**

- **Amapita kuti?**
- **Amafunsa kwa ndani?**
- **Amayamba kufunsa pakapita nthawi yayitali bwanji?**
- **Amafunsa ndani popanga chiganizo choterechi?**
- **Amakhudzidwa ndi ndani popanga chiganizocho?**
- **Kodi zimasiyana akakhala matenda ena?**

1. In general, what are the communities’ in which you works perceptions of formal health care?
   - - How does this vary between public and private providers?
     - How does this vary based on the type of service?

**Kodi anthu kunoko chithandizo chomwe chimaperekedwa kuchipatala amachiona bwanji?**

- **Nanga pali kusiyana pakati pa zipatala za boma ndi zipatala zolipira?**
- **Nanga zimasiyana bwanji? (kutengera ndi mtundu wa thandizo lomwe amapereka?)**

1. Over the last 2 years, what changes have you seen in the choice of providers available to the communities in which you work?
   - - What can you say about the quality of the services that are provided?

**Kodi mu zaka ziwiri zapitazi pali kusintha kwina kuli konse pa za komwe anthu amapita kukapezako thandizo la zaumoyo?**

- **Munganeneko chiyani pa zakolite ya thandizo lomwe limaperekedwa kumeneko?**

1. What would you say are the main challenges faced by people in the communities where you work when seeking health care?

PROMPT:

- How does this differ between formal and informal health care?
- How does this differ between the public and private sector?
- Would you suggest solutions to various challenges people face to access health services?

**Nanga ndi mavuto otani omwe anthu akuno amakumana nawo pokafuna thandizo kuchipatala?**

- **Pali kusiyana kwanji pakati pa ku chipatala ndi thandizo lina ie kwa asing’anga**
- **Pali kusiyana pakati pa zipatala za boma ndi zipatala zolipira**
- **Mukuona ngati tingatani pothetsa mavutowa?**

1. What challenges do you face in providing health care services?

PROMPT:

- Drug stock outs, lack of equipment, lack of knowledge, lack of referral systems
- Which of these problems do you have in relation to lung health?

**Kodi mumakumana ndi mavuto otani popereka thandizo?**

**Funsitsani za**

- **TB, chifuwa cha befu, mphumu,**
- **Kupereka Thandizo kwa anthu olumala**
- **Kusowa kwa mankhwala, zipangizo**
- **Kusadziwa kapena kusowa ukadaulo (pa za TB, chifuwa cha befu, mphumu)**
- **Mavuto potumiza odwala kuchipatala**
- **Pamavuto omwe mwatchulawa, ndi mavuto anji omwe ndi okhudzana ndi matenda omwe amagwira mapapo?**

***Summarise key points from pathways to care seeking discussion.***

***Thank you very much for providing me with information on your knowledge of care seeking behaviour, unless you want to add anything in this area, I would like to ask you some more questions about informal health providers in the communities where you work.***

### Perceptions of Informal Health Care Providers (NB: HSAs are not IHPs)

1. What do you understand by the term informal health provider?

**Kodi mumamvetsa kuti mavolontiya a zaumoyo kumudzi ndi ndani?**

1. Can you tell us the different IHPs that are in the community where you work? (*Instruction to the researcher: ask the below in relation to each of the IHPs mentioned.)*

What services do they offer?

- - Information (what diseases?), referrals etc.
  - Do people use these services? Why/why not?
- What additional services could they provide that they are not already?
- What could be done to improve the quality of the services they provide?

**Kodi ndi magulu ati a mavolontiya a zaumoyo omwe alipo kudera lino?**

- **Amapereka thandizo lanji?**
  - **Pa matenda anji? Amatha kutumizaodwala kuchipatala (referral)?**
  - **Nanga anthu amagwiritsa ntchito upangiri wawo? Fotokozani?**
- **Kupatula ntchito zomwe akugwira mavololontiya a za umoyo a kumudziwa, pali ntchito zina zomwe angagwirenso zokhudzana ndi zaumoyo? Fotokozani?**
- **Pachitike chiyani kuti iwowa azipereka thandizo labwino (kolite)?**

1. Do you work with IHPs?

If yes, in what ways do you work with them? Explain

- - What are the advantages of working with IHPs?
  - What are the challenges of working with IHPs?
    - How could these challenges be overcome?
- If no, in the future how do you think health care providers could work with IHPs?

**Kodi mumagwira ntchito ndi mavolontiya a za umoyo kapena ma komiti amm’idzi?**

**Mumagwira nawo bwanji?**

- **Pali ubwino wanji wogwira ndi magulu amenewa?**
- **Nanga kuipa kwake ndi kotani? Mavuto ogwira ntchito ndi magulu amenewa ndi otani?**
- **Mavuto amenewa angathetsedwe bwanji?**

**Ngati sagwira ntchito ndi ndi magulu akumudzi, mukuganiza kuti mtsogolo muno mungadzagwire nawo bwanji?**

1. Who are the target populations of these IHPs?
   - Are there any groups in your community or neighbouring communities left out of access to IHP services?
   - If so which are these groups?
   - Explain?

**Kodi ntchito zawo amafikira nazo ndani?**

- **Kodi alipo magulu ena a anthu mdera lino kapena madera oyandikana nawo omwe safikiridwa ndi thandizo loperekedwa ndi mavolontiya amenewa?**
- **Ngati alipo, ndiati? Chifukwa chiyani? Fotokozani.**

1. What is your perception of IHPs and the services they offer?

**Kodi thandizo lomwe mavolontiya a zaumoyo amm’idzi amapereka lino mumaliwona bwanji?**

- - Are there cases in your community where IHPs’ services are shunned? Explain? – Prompt on the status of the provider i.e HIV, economic status, education levels etc

**Kodi zimachitika kuti thandizo lomwe mavolontiya a za umoyo amapereka limakanidwa? Fotokozani.**

**Funsitsanipotengera:**

- **maonekedwe,**
- **maphunziro**
- **kupatakwamunthu,**
- **kasungachinsinsi**
- **mtundu wa munthu**
- **kolite ya chithandizo chomwe amapereka**

***Summarise key points from IHP discussion.***

***Thank you very much for providing me with information on your knowledge of IHPs, unless you want to add anything in this area, I would like to ask you some more questions about disability in the communities where you work.***

***Is there anything you would like to add regarding any of the topics we have discussed? Many thanks for your time today it has been most useful to our study.***

## Focus Group Discussion Guide for Village Health Committee Members

Prior to the focus group beginning I would just like to ask you a few demographic questions. ***(These questions should be asked of the participant prior to being in the main focus group as the questions are personal)***

### Demographics

**What is your educational level?**

**What do you do to earn an income?**

**Are you male or female?**

**How old are you?**

**What is your religion?**

**What is your tribe?**

### Introduction to the Focus Group

Thank you for taking the time to meet with me today. I hope you will think of this focus group as an opportunity to share your communities’ experiences. I hope that you feel able to express your opinions openly and honestly. Within the focus group I will ask you a series of questions around different topic areas. Firstly, I would like to ask you a few questions about your knowledge of chronic cough and breathlessness

### Knowledge of Lung Health

1. When would you call a cough a chronic one?

PROMPT:

- How long would someone have to have been coughing before you would say it was chronic?

**Kodi chifuwa mumachitcha chamgonagona pakapita nthawi yayitali bwanji?**

**(Funsitsani za nthawi yomwe munthuyo wakhala akukhosomola)**

1. What diseases do you know are associated with chronic cough?

PROMPT:

- TB, Asthma, Other CAD?

**Kodi ndi matenda anji omwe amagwirizana ndi chifuwa chamgonagona? Funsitsani za mphumu kapenanso befu (chifuwa chotha phuma) (wheezing)**

1. How would you describe breathlessness or wheezing?

**Kodi befu ndi chiyani?**

1. What diseases do you know that are associated with difficulty in breathing?

PROMPT:

- - TB, Asthma, other CAD?

**Kodi ndi matenda anji omwe amakhudzana ndi befu**

**Funsitsani za**

- **TB, Asthma ndi CAD**

1. Would you tell us what you know about XX? (*Instruction to researcher: XX refers to each of the diseases of interest that have been mentioned in question 2. You should ask about them in order: TB, Asthma,CAD.)*

PROMPT:

- What are the causes of these diseases?
- What are the signs and symptoms?
  - Is this disease associated with shortness of breath?
    - If yes, how do you tell if someone has shortness of breath?
- How are they transmitted?
- How are they prevented?
- How do you know if someone has this disease?
- What is the treatment for these diseases?

**Mungatiuzeko zomwe mukudziwa za XX (*Instruction to researcher: XX refers to each of the diseases of interest that have been mentioned in question 2. You should ask about them in order: TB, Asthma, CAD.)***

- - - **Zomwe zimayambitsa matenda amenewa**
    - **Zizindikiro zake ndi zotani?**
      - **Kodi matenda amenewa amakhudzana ndi befu?**
      - **Mungadziwe bwanji kuti munthu ali ndi befu?**
    - **Kufala kwake**
    - **Kupewa kwake**
    - **Mungadziwe bwanji kuti munthu ali ndi matenda amenewa?**
    - **Mankhwala amatenda amenewa ndi chiyani?**

1. Where do you get the information you have shared with me about lung health?

**Kodi zimene mwafotokozazi zokhudzana ndi matenda omwe amagwira mapapo mumazimvera kuti?**

PROMPT:

- Within your community, are there groups that provide information about lung health?
  - Can you tell me who they are? Name them?

**Kodi mdera/mmudzi muno ndi magulu ati omwe amafalitsa uthenga wokhudzana ndi matenda omwe amagwira mapapo (ie TB chronic cough)?**

**Ndi ndani?**

- - 1. If so, how do they provide this information?
       1. Drama, song, leaflet, talk, poster, radio etc.

**Ngati alipo, amafalitsa motani( ieDrama, song, leaflet, talk, poster, radio)?**

- - 1. Which group gives a clearer understanding of lung health issues? Explain?

**Mwamagulu omwe mwatchulawa ndimagulu ati omwe amafalitsa mauthengawa momveka bwino kwambiri? Fotokozani?**

- - 1. Which method(s) gives a clearer understanding of lung health issues? Explain?

**Ndi gulu liti komanso ndi njira ziti zimene zimapereka uthengawu momveka bwino kwambiri pa nkhani yamatenda omwe amagwira mapapo? Fotokozani?**

1. What recommendations can you give to improve communication on health information focusing on lung health?

**Munganenepo zotani pofuna kupititsa mtsogolo kafalitsidwe kamauthenga okhudzana ndi matenda omwe amagwira mapapo (TB, asthma komansochifuwa cha mgonagona)**

PROMPT:

- How would you like to receive this information in the future?

**Mungakonde mutamaulandira bwanji uthenga umenewu mtsogolo muno?**

- Is there any specific areas that you would like additional information on?

**Pali zinthu zina zimene mungafune uthenga wina owonjezera?**

***Summarise key points from knowledge discussion.***

***Thank you very much for providing me with information on your knowledge of lung health, unless you want to add anything in this area, I would like to ask you some more questions about how people seek health care when they get sick in this community.***

### Pathways to Care Seeking

1. What do people in this community do when they get sick?

**Kodi kunoko anthu akadwala amatani?**

PROMPT:

- What type of care do they seek?
  - Self treatment, traditional methods, formal health care, faith healing
- **Kodi amakafuna thandizo kuti? Funsitsani za kupeza thandizo kwa asing’anga, kuchipatala, mapemphero komanso kuzithandiza okha pakhomo.**
  - Why this type of care?
- **Chifukwa chiyani amasankha kutero?**
- How do they decide to seek care?

**Kodi ndi ndani amapanga ganizo lopita kukafuna thandizolo?**

- - Who is involved in the decision?

**Ndi anthu ati omwe amakhudzidwa ndi kupanga chiganizocho?**

- - What factors are important to consider in the decision to seek care?
    - E.g. accessibility, distance, cost

**Ndi zinthu ziti zomwe anthu amaona akafuna kupanga chiganizo cha kokalandira thandizo la za umoyo?**

**2.** If someone has a chronic cough in this community, what do they do?

PROMPT:

- Where do they go?
- Who do they speak to?
- When do they do this?
- Who is involved in this?
- How is this different to when people get sick with other diseases?

**Kodi mdera lino, munthu akakhala ndi chifuwa cha mgonagona amatani?**

- **Amapita kuti kukafuna thandizo la zaumoyo**
- **Amayamba kufuna thandizo pakapita nthawi yayitali bwanji?**
- **Amafunsa ndani popanga chiganizo choterechi?**
- **Amakhudzidwa ndi ndani popanga chiganizocho?**
- **Kodi zimasiyana akakhala matenda ena?**

**3.** If someone has difficulty breathing in this community, what do they do?

PROMPT:

- Where do they go?
- Who do they speak to?
- When do they do this?
- Who is involved in this?
- How is this different to when people get sick with other diseases?

**Nanga munthu akakhala ndi befu amatani?**

- **Amapitakuti?**
- **Amafunsakwandani?**
- **Amayambakufunsapakapitanthawiyayitalibwanji?**
- **Amafunsa ndani popanga chiganizo choterechi?**
- **Amakhudzidwa ndi ndani popanga chiganizocho?**
- **Kodi zimasiyana akakhala matenda ena?**

**4.** In general, what are the communities’ perceptions of formal health care?

- - 1. How does this vary between public and private providers?
    2. How does this vary based on the type of service?

**Kodi anthu kunoko chithandizo chomwe chimaperekedwa ku chipatala amachiona bwanji?**

- **Nanga pali kusiyana pakati pa zipatala za boma ndi zipatala zolipira?**
- **Nanga zimasiyana bwanji? (kutengera ndi mtundu wathandizo lomwe amapereka?)**

**5.** What would you say are the main challenges faced by people in this community when seeking health care?

PROMPT:

- How does this differ between formal and informal health care?
- How does this differ between the public and private health facilities?
- Would you suggest solutions to various challenges people face to access health services?

**Nanga ndi mavuto otani omwe anthu akuno amakumana nawo pokafuna thandizo ku chipatala?**

- **Pali kusiyana kwanji pakati pa kuchipatala ndi thandizo lina ie kwa asing’anga**
- **Pali kusiyana pakati pa zipatala za bomandi zipatala zolipira**
- **Mukuona ngati tingatani pothetsa mavutowa?**

6. Over the last 2 years, what changes have you seen in the choice of providers available in your community?

- What can you say about the quality of the services that are provided?

**Kodi mu zaka ziwiri zapitazi pali kusintha kwina kuli konse pa za komwe anthu amapita kukapezako thandizo la za umoyo?**

- **Munganeneko chiyani pa za kolite yathandizo lomwe limaperekedwa kumeneko?**

***Summarise key points from pathways to care seeking discussion.***

***Thank you very much for providing me with information on your knowledge of care seeking behaviour, unless you want to add anything in this area, I would like to ask you some more questions about informal health providers in your community.***

### Perceptions of Informal Health Care Providers (NB: HSAs are not IHPs)

1. What do you understand by the term informal health provider?

**Kodi mumamvetsa kuti mavolontiya a zaumoyo kumudzi ndi ndani?**

1. Can you tell us the different IHPs that are in this community? **(*Instruction to the researcher: ask the below in relation to each of the IHPs mentioned.)***

- What services do they offer?
  - Information (what diseases?), referrals etc.
  - Do people use these services? Why/why not?
- What additional services could they provide that they are not already?
- What could be done to improve the quality of the services they provide?

**Kodi ndi magulu ati a mavolontiya a zaumoyo omwe alipo kudera lino?**

- **Amapereka thandizo lanji?**
  - **Pa matenda anji? Amatha kutumiza odwala ku chipatala (referral)?**
  - **Nanga anthu amagwiritsa ntchito upangiri wawo? Fotokozani?**
- **Kupatula ntchito zomwe akugwira mavololontiya a zaumoyo akumudziwa, pali ntchito zina zomwe angagwirenso zokhudzana ndi zaumoyo? Fotokozani?**
- **Pachitike chiyani kuti iwowa azipereka thandizo labwino (kolite)?**

1. Who are the target populations of these IHPs?
   - Are there any groups in your community or neighbouring communities left out of access to IHP services?
   - If so which are these groups?
   - Explain?

**Kodi ntchito zawo amafikira nazo ndani?**

- **Kodi alipo magulu ena a anthu mdera lino kapena madera oyandikana nawo omwe safikiridwa ndi thandizo loperekedwa ndi mavolontiya amenewa?**
- **Ngatialipo, ndiati? Chifukwa chiyani? Fotokozani.**

1. What is your perception of IHPs and the services they offer?

**Kodi thandizo lomwe mavolontiya a zaumoyo amm’idzi amapereka lino mumaliwona bwanji?**

- - Are there cases in your community where IHPs’ services are shunned? Explain? – Prompt on the status of the provider i.e HIV, economic status, education levels, tribe etc
- **Kodi zimachitika kuti thandizo lomwe mavolontiya a za umoyo amapereka limakanidwa? Fotokozani.**

**Funsitsani potengera:**

- **maonekedwe,**
- **maphunziro**
- **kupata kwa munthu,**
- **kasunga chinsinsi**
- **mtundu wa munthu**
- **kolite ya chithandizo chomwe amapereka**

***Summarise key points from IHP discussion.***

***Thank you very much for providing me with information on your knowledge of IHPs, unless you want to add anything in this area, I would like to ask you some more questions about disability your community.***

## Interview Guide for Health Care Workers

Thank you for taking the time to meet with me today. I hope you will think of this interview as an opportunity to share your experiences. I hope that you feel able to express your opinions openly and honestly. Within the interview I will ask you a series of questions based around different topic areas. Firstly, I would like to ask you a few demographic questions**.**

### Demographics

**What is your educational level?**

**What are your main roles and responsibilities?**

**Are you male or female?**

**How old are you?**

**For how long have you been working as a health care worker?**

### Knowledge of TB and Chronic Cough

1. When do you call a cough a chronic one?

PROMPT:

- How long would someone have to have been coughing before you would say it was chronic?

**Kodi chifuwa mumachitcha cha mgonagona patapita nthawi yayitali bwanji? (funsitsani za nthawi yomwe munthuyo wakhala akukhosomola)**

1. What diseases do you know are associated with chronic cough?

PROMPT:

- Asthma, TB, other CAD?

**Kodi ndi matenda anji omwe amagwirizana ndi chifuwa chamgonagona? Funsitsani za mphumu kapenanso befu (chifuwa chotha phuma)**

1. How would you describe breathlessness or wheezing?

**Kodi befu ndi chiyani?**

1. What diseases do you know that are associated with difficulty in breathing?

PROMPT:

- TB, Asthma, other CAD?

**Kodi ndi matenda anji omwe amakhudzana ndi befu**

**Funsitsani za**

- **TB, Asthma, other CAD**

1. Would you tell us what you know about X? (*Instruction to researcher: X refers to each of the diseases of interest that have been mentioned in question 2 and 4. You should ask about them in order: TB, Asthma, and other CAD.)*

PROMPT:

- What are the causes of these diseases?
- What are the signs and symptoms?
  - Is this disease associated with shortness of breath?
    - If yes, how do you tell if someone has shortness of breath?
- How are they transmitted?
- How are they prevented?
- How do you know if someone has this disease?
- What is the treatment for these diseases?

**Mungatiuzeko zomwe mukudziwa za XX (*Instruction to researcher: XX refers to each of the diseases of interest that have been mentioned in question 2. You should ask about them in order: TB, Asthma, CAD.)***

- - - **Zomwe zimayambitsa matenda amenewa**
    - **Zizindikiro zake ndi zotani?**
      - **Kodi matenda amenewa amakhudzana ndi befu?**
      - **Mungadziwe bwanji kuti munthu ali ndi befu?**
    - **Kufala kwake**
    - **Kupewa kwake**
    - **Mungadziwe bwanji kuti munthu ali ndi matenda amenewa?**
    - **Mankhwala amatenda amenewa ndi chiyani?**

1. Where did you get the information you have shared with me about lung health?

**Kodi zimene mwafotokozazi zokhudzana ndi matenda omwe amagwira mapapo mumazimvera kuti?**

PROMPT:

- Within the catchment area of this facility, are there groups that provide information about lung health?
  - Can you tell me who they are? Name them?

**Kodi mdera lozungulira chipatala chino muli magulu/anthu omwe amafalitsa uthengawa? (TB ndi Chronic cough)?**

**Ndi ndani?**

- - 1. If so, how do they provide this information?
       1. Drama, song, leaflet, talk, poster, radio etc.

**Ngati alipo, amafalitsa motani?**

- - 1. In your opinion, which group gives a clearer understanding of lung health issues? Explain?

**Mwa magulu mwatchulawa ndi magulu ati omwe amafalitsa mauthengawa momveka bwino kwambiri?**

**Fotokozani?**

- - 1. Which method(s) gives a clearer understanding of lung health issues? Explain?

**Ndi njira ziti zimene zimapereka uthengawu momveka bwino kwambiri?**

**Fotokozani?**

1. What recommendations can you give to improve communication on health information focusing on lung health?

**Munganenepo zotani pofuna kupititsa mtsogolo kafalitsidwe ka mauthenga okhudzana ndi matenda omwe amagwira mapapo (TB, asthma komanso chifuwa cha mgonagona)**

PROMPT:

- What are the specific areas that you would like to know more about?

**Pali zinthu zina zimene mungafune uthenga wina owonjezera?**

- How would you like to receive this information in the future?

**Mungakonde mutamaulandira bwanji uthenga umenewu mtsogolo muno?**

***Summarise key points from knowledge discussion.***

***Thank you very much for providing me with information on your knowledge of lung health, unless you want to add anything in this area, I would like to ask you some more questions about how people seek health care when they get sick in the communities that this facility serves.***

### Pathways to Care Seeking

1. What do people do when they get sick?

**Kodi kunoko anthu akadwala amatani?**

PROMPT:

- What type of care do they seek?
  - Self treatment, traditional methods, formal health care, faith healing

**Kodi amakafuna thandizo kuti? Funsitsani za kupeza thandizo kwa asing’anga, ku chipatala, mapemphero komanso kuzithandiza okha pakhomo.**

- - Why this type of care?
- **Chifukwa chiyani amasankha kutero?**
- How do they decide to seek care?

**Kodi ndi ndani amapanga ganizo lopita kukafuna thandizolo?**

- - Who is involved in the decision?

**Ndi anthu ati omwe amakhudzidwa ndi kupanga chiganizocho?**

- - What factors are important to consider in the decision to seek care?
    - E.g. accessibility, distance, cost

**Ndi zinthu ziti zomwe anthu amaona akafuna kupanga chiganizo cha kokalandira thandizo la za aumoyo?**

1. If someone has a chronic cough in this community, what do they do?

PROMPT:

- Where do they go?
- Who do they speak to?
- When do they do this?
- Who is involved in this?
- How is this the same as when people get sick with other diseases?

**Kodi mdera lino, munthu akakhala ndi chifuwa cha mgonagona amatani?**

- **Amapita kuti kukafuna thandizo la zaumoyo**
- **Amayamba kufuna thandizo pakapita nthawi yayitali bwanji?**
- **Amafunsa ndani popanga chiganizo choterechi?**
- **Amakhudzidwa ndi ndani popanga chiganizocho?**
- **Kodi zimasiyana akakhala matenda ena?**

1. If someone has difficulty breathing in this community, what do they do?

PROMPT:

- Where do they go?
- Who do they speak to?
- When do they do this?
- Who is involved in this?
- How is this the same as when people get sick with other things?
- How is this different to when people get sick with other things?

**Nanga munthu akakhala ndi befu amatani?**

- **Amapita kuti?**
- **Amafunsa kwa ndani?**
- **Amayamba kufunsa pakapita nthawi yayitali bwanji?**
- **Amafunsa ndani popanga chiganizo choterechi?**
- **Amakhudzidwa ndi ndani popanga chiganizocho?**
- **Kodi zimasiyana akakhala matenda ena?**

1. In general, what are the communities’ perceptions of formal health care?
   - 1. How does this vary between public and private providers?
     2. How does this vary based on the type of service?

**Kodi anthu kunoko chithandizo chomwe chimaperekedwa kuchipatala amachiona bwanji?**

- **Nanga pali kusiyana pakati pa zipatala za boma ndi zipatala zolipira?**
- **Nanga zimasiyana bwanji? (kutengera ndi mtundu wa thandizo lomwe amapereka?)**

1. Over the last 2 years, what changes have you seen in the choice of providers available to the communities that this facility serves?
   - 1. What can you say about the quality of the services that are provided?

**Kodi mu zaka ziwiri zapitazi pali kusintha kwina kuli konse pa za komwe anthu amapita kukapezako thandizo la zaumoyo?**

- **Munganeneko chiyani pa zakolite ya thandizo lomwe limaperekedwa kumeneko?**

1. What would you say are the main challenges faced by people in the communities where you work when seeking health care?

PROMPT:

- How does this differ between formal and informal health care?
- How does this differ between the public and private sector?
- Would you suggest solutions to various challenges people face to access health services?

**Nanga ndi mavuto otani omwe anthu akuno amakumana nawo pokafuna thandizo kuchipatala?**

- **Pali kusiyana kwanji pakati pa ku chipatala ndi thandizo lina ie kwa asing’anga**
- **Pali kusiyana pakati pa zipatala za boma ndi zipatala zolipira**
- **Mukuona ngati tingatani pothetsa mavutowa?**

1. What challenges do you face in providing health care services?

PROMPT:

- Drug stock outs, lack of equipment, lack of knowledge, lack of referral systems
- Which of these problems do you have in relation to treating lung health?

**Kodi mumakumana ndi mavuto otani popereka thandizo?**

**Funsitsani za**

- **TB, chifuwa cha befu, mphumu,**
- **Kupereka Thandizo kwa anthu olumala**
- **Kusowa kwa mankhwala, zipangizo**
- **Kusadziwa kapena kusowa ukadaulo (pa za TB, chifuwa cha befu, mphumu)**
- **Mavuto potumiza odwala kuchipatala**
- **Pamavuto omwe mwatchulawa, ndi mavuto anji omwe ndi okhudzana ndi matenda omwe amagwira mapapo?**

***Summarise key points from pathways to care seeking discussion.***

***Thank you very much for providing me with information on your knowledge of care seeking behaviour, unless you want to add anything in this area, I would like to ask you some more questions about informal health providers in the communities where you work.***

### Perceptions of Informal Health Care Providers (NB: HSAs are not IHPs)

1. What do you understand by the term informal health provider?

**Kodi mumamvetsa kuti mavolontiya a zaumoyo kumudzi ndi ndani?**

1. Can you tell us the different IHPs that are in the community that this facility serves? (*Instruction to the researcher: ask the below in relation to each of the IHPs mentioned.)*

What services do they offer?

- - Information (what diseases?), referrals etc.
  - Do people use these services? Why/why not?
- What additional services could they provide that they are not already?
- What could be done to improve the quality of the services they provide?

**Kodi ndi magulu ati a mavolontiya a zaumoyo omwe alipo kudera lino?**

- **Amapereka thandizo lanji?**
  - **Pa matenda anji? Amatha kutumizaodwala kuchipatala (referral)?**
  - **Nanga anthu amagwiritsa ntchito upangiri wawo? Fotokozani?**
- **Kupatula ntchito zomwe akugwira mavololontiya a za umoyo a kumudziwa, pali ntchito zina zomwe angagwirenso zokhudzana ndi zaumoyo? Fotokozani?**
- **Pachitike chiyani kuti iwowa azipereka thandizo labwino (kolite)?**

1. Do you work with IHPS?

- If yes, in what ways do you work with them? Explain
  - What are the advantages of working with IHPs?
  - What are the challenges of working with IHPs?
    - How could these challenges be overcome?
- If no, in the future how do you think health care providers could work with IHPs?

**Kodi mumagwira ntchito ndi mavolontiya a za umoyo kapena ma komiti amm’idzi?**

**Mumagwira nawo bwanji?**

- **Pali ubwino wanji wogwira ndi magulu amenewa?**
- **Nanga kuipa kwake ndi kotani? Mavuto ogwira ntchito ndi magulu amenewa ndi otani?**
- **Mavuto amenewa angathetsedwe bwanji?**

**Ngati sagwira ntchito ndi ndi magulu akumudzi, mukuganiza kuti mtsogolo muno mungadzagwire nawo bwanji?**

1. Who are the target populations of these IHP?
   - Are there any groups in your community or neighbouring communities left out of access to IHP services?
   - If so which are these groups?
   - Explain?

**Kodi ntchito zawo amafikira nazo ndani?**

- **Kodi alipo magulu ena a anthu mdera lino kapena madera oyandikana nawo omwe safikiridwa ndi thandizo loperekedwa ndi mavolontiya amenewa?**
- **Ngati alipo, ndiati? Chifukwa chiyani? Fotokozani.**

1. What is your perception of IHPs and the services they offer?

**Kodi thandizo lomwe mavolontiya a zaumoyo amm’idzi amapereka lino mumaliwona bwanji?**

- - Are there cases in your community where IHPs’ services are shunned? Explain? – Prompt on the status of the provider i.e HIV, economic status, education levels etc

**Kodi zimachitika kuti thandizo lomwe mavolontiya a za umoyo amapereka limakanidwa? Fotokozani.**

**Funsitsanipotengera:**

- **maonekedwe,**
- **maphunziro**
- **kupatakwamunthu,**
- **kasungachinsinsi**
- **mtundu wa munthu**
- **kolite ya chithandizo chomwe amapereka**

***Summarise key points from IHP discussion.***

***Thank you very much for providing me with information on your knowledge of IHPs, unless you want to add anything in this area, I would like to ask you some more questions about disability in the communities where you work.***

***Is there anything you would like to add regarding any of the topics we have discussed? Many thanks for your time today it has been most useful to our study.***

## Key informant interview guide for Traditional Leaders

Thank you for taking the time to meet with me today. I hope you will think of this interview as an opportunity to share your experiences. I hope that you feel able to express your opinions openly and honestly. Within the interview I will ask you a series of questions based around different topic areas. Firstly, I would like to ask you a few demographic questions**.**

### Demographics

**What do you do to earn an income?**

**What is your educational level?**

**Are you male or female?**

**What is your religion?**

**What is your tribe?**

### Knowledge of Lung Health

**1.** Within your community are there groups/individuals that provide information about lung health?

**Kodi mdera/mmudzi muno alipo magulu omwe amafalitsa uthenga wokhudzana ndi matenda omwe amagwira mapapo (ie TB chronic cough)?**

**Ndi ndani?**

- - 1. If so, how do they provide this information?
       1. Drama, song, leaflet, talk, poster, radio etc.

**Ngati alipo, amafalitsa motani (ie Drama, songs, leaflets, talk, poster, radio)?**

- - 1. Which group gives a clearer understanding of lung health issues? Explain?

**Mwamagulu omwe mwatchulawa ndi magulu ati omwe amafalitsa mauthengawa momveka bwino kwambiri? Fotokozani?**

- - 1. Which method(s) gives a clearer understanding of lung health issues? Explain?

**Ndi njira ziti zimene zimapereka uthengawu momveka bwino kwambiri pa nkhani yamatenda omwe amagwira mapapo?Fotokozani?**

1. What recommendations can you give to improve communication on health information focusing on lung health?

**Munganenepo zotani pofuna kupititsa mtsogolo kafalitsidwe kamauthenga okhudzana ndi matenda omwe amagwira mapapo (TB, asthma komanso chifuwa cha mgonagona)**

PROMPT:

- What else would you like to know about these diseases?

**Mukanakonda mukanadziwa zotani za matenda amenewa?**

- How would you like to receive this information in the future?

**Mungakonde mutamaulandira bwanji uthenga umenewu mtsogolo muno?**

- Is there any specific areas that you would like additional information on?

**Pali zinthu zina zimene mungafune uthenga wina owonjezera?**

***Summarise key points from knowledge discussion.***

***Thank you very much for providing me with information on your knowledge of chronic cough, unless you want to add anything in this area, I would like to ask you some more questions about how people seek health care when they get sick in this community.***

### Pathways to Care Seeking

1. What do people in this community do when they get sick?

**Kodi kunoko anthu akadwala amatani?**

PROMPT:

- What type of care do they seek?
  - Self treatment, traditional methods, formal health care, faith healing

**Kodi amakafuna thandizo kuti? Funsitsani za kupeza thandizo kwa asing’anga, ku chipatala, mapemphero komanso kuzithandiza okha pakhomo.**

- - Why this type of care?

**Chifukwa chiyani amasankha kutero?**

- How do they decide to seek care?

**Kodi amatani popanga chiganizo kupita kukafuna thandizolo?**

- - Who is involved in the decision?

**Ndi anthu ati omwe amakhudzidwa ndi kupanga chiganizocho?**

- - What factors are important to consider in the decision to seek care?
    - E.g. accessibility, distance, cost

**Ndi zinthu ziti zomwe anthu amaona akafuna kupanga chiganizo cha kokalandira thandizo la za aumoyo?**

1. If someone has a chronic cough in this community, what do they do?

PROMPT:

- Where do they go?
- Who do they speak to?
- When do they do this?
- Who is involved in this?
- How is this different to when people get sick with other diseases?

**Kodi mdera lino, munthu akakhala ndi chifuwa cha mgonagona amatani?**

- **Amapita kuti kukafuna thandizo la zaumoyo**
- **Amayamba kufuna thandizo pakapita nthawi yayitali bwanji?**
- **Amafunsa ndani popanga chiganizo choterechi?**
- **Amakhudzidwa ndi ndani popanga chiganizocho?**
- **Kodi zimasiyana bwanji akakhala kuti ndi matenda ena?**

1. If someone has difficulty breathing in this community, what do they do?

PROMPT:

- Where do they go?
- Who do they speak to?
- When do they do this?
- Who is involved in this?
- How is this different to when people get sick with other things?

**Nanga munthu akakhala ndi befu amatani?**

- **Amapita kuti?**
- **Amafunsa kwa ndani?**
- **Amayamba kufunsa pakapita nthawi yayitali bwanji?**
- **Amafunsa ndani popanga chiganizo choterechi?**
- **Amakhudzidwa ndi ndani popanga chiganizocho?**
- **Kodi zimasiyana bwanji akakhala kuti ndi matenda ena?**

1. In general, what are the communities’ perceptions of formal health care?

**Kodi anthu kunoko chithandizo chomwe chimaperekedwa kuchipatala amachiona bwanji?**

- - 1. How does this vary between public and private providers?

**Nanga palikusiyana kwanji pakati pa zipatala za boma ndi zipatala zolipira?**

- - 1. How does this vary based on the type of service?

**Nanga zimasiyana bwanji? (kutengera ndi mtundu wa thandizo lomwe amapereka?)**

1. Over the last 2 years, what changes have you seen in the choice of providers available in your community?
   - 1. What can you say about the quality of the services that are provided?

**Kodi mu zaka ziwiri zapitazi pali kusintha kwina kuli konse pa zakomwe anthu amapita kukapezako thandizo la zaumoyo?**

- **Munganeneko chiyani pa za kolite ya thandizo lomwe limaperekedwa kumeneko?**

1. What would you say are the main challenges faced by people in this community when seeking health care?

PROMPT:

- How does this differ between formal and informal health care?
- How does this differ between the public and private sector?
- Would you suggest solutions to various challenges people face to access health services?

**Nanga ndimavutootaniomweanthuakunoamakumananawopokafunathandizokuchipatala?**

- **Pali kusiyana kwanji pakati pa kuchipatala ndi thandizo lina ie kwa asing’anga**
- **Pali kusiyana pakati pa zipatala za boma ndi zipatala zolipira**
- **Mukuona ngati tingatani pothetsa mavutowa?**

***Summarise key points from pathways to care seeking discussion.***

***Thank you very much for providing me with information on your knowledge of care seeking behaviour, unless you want to add anything in this area, I would like to ask you some more questions about how people seek health care when they get sick in the communities where you work informal health providers in the communities where you work.***

### Perceptions of Informal Health Care Providers (NB: HSAs are not IHPs)

1. What do you understand by the term informal health provider?

**Kodi mumamvetsa kuti mavolontiya a zaumoyo kumudzi ndi ndani?**

1. Can you tell us the different IHPs that are in this community? (*Instruction to the researcher: ask the below in relation to each of the IHPs mentioned.)*

What services do they offer?

- - Information (what diseases?), referrals etc.
  - Do people use these services? Why/why not?
- What additional services could they provide that they are not already?
- What could be done to improve the quality of the services they provide?

**Kodi ndi magulu ati a mavolontiya a zaumoyo omwe alipo kudera lino?**

- **Amapereka thandizo lanji?**
  - **Pa matenda anji? Amatha kutumiza odwala ku chipatala (referral)?**
  - **Nanga anthu amagwiritsa ntchito upangiri wawo? Fotokozani?**
- **Kupatula ntchito zomwe akugwira mavololontiya a zaumoyo a kumudziwa, pali ntchito zina zomwe angagwirenso zokhudzana ndi zaumoyo? Fotokozani?**
- **Pachitike chiyani kuti iwowa azipereka thandizo labwino (kolite)?**

1. Who are the target populations of these IHP?
   - Are there any groups in your community or neighbouring communities left out of access to IHP services?
   - If so which are these groups?
   - Explain?

**Kodi ntchito zawo amafikira nazo ndani?**

- **Kodi alipo magulu ena a anthu mdera lino kapena madera oyandikana nawo omwe safikiridwa ndi thandizo loperekedwa ndi mavolontiya amenewa?**
- **Ngati alipo, ndiati? Chifukwa chiyani? Fotokozani.**

1. What is your perception of IHPs and the services they offer?

**Kodi thandizo lomwe mavolontiya a zaumoyo amm’idzi amapereka mdera lino mumaliwona bwanji?**

- - Are there cases in your community where IHPs’ services are shunned? Explain? – Prompt on the status of the provider i.e HIV, economic status, education levels, tribe etc
- **Kodi zimachitika kuti thandizo lomwe mavolontiya a za umoyo amapereka limakanidwa? Fotokozani.**

**Funsitsani potengera:**

- **maonekedwe,**
- **maphunziro**
- **kupatakwamunthu,**
- **kasungachinsinsi**
- **mtundu wa munthu**
- **kolite ya chithandizo chomwe amapereka**

***Summarise key points from IHP discussion.***

***Thank you very much for providing me with information on your knowledge of IHPs, unless you want to add anything in this area, I would like to ask you some more questions about disability in the communities where you work.***

***Is there anything you would like to add regarding any of the topics we have discussed? Many thanks for your time today it has been most useful to our study.***

## In-depth Interview Guide for Patient with TB or CAD

Thank you for taking the time to meet with me today. I hope you will think of this interview as an opportunity to share your experiences. I hope that you feel able to express your opinions openly and honestly. Within the interview I will ask you a series of questions based around different topic areas. Firstly, I would like to ask you a few demographic questions**.**

### Demographics

**How old are you?**

**Are you male or female?**

**What is their highest level of education?**

**Do you work?**

**If yes, what do you do?**

**What is your religion?**

**What is your tribe?**

Have you been diagnosed with one of the following diseases?

**Kodi achipatala adakupezaniko ndi matenda awa:**

- Asthma
- TB
- Chronic Obstructive Pulmonary Diseases (COPD)
- Bronchitis **Chifuwa cha mgonagona**
- Pneumonia **Chibayo**

***Thank you very much for providing me with your demographic information. I would like to ask you some screening questions about your general health and wellbeing.***

### Washington Group Questions and Perceptions of Disability

Because of a health condition, do youhave difficulty or problem in the following:

| - - - 1. Seeing even if wearing glasses?   **(Kodi mumavutika kuwona ngakhale mutavala magalasiamaso?)** | 1. No - no difficulty 2. Yes - some difficulty 3. Yes - a lot of difficulty 4. Cannot see at all |  |
| --- | --- | --- |
| - - - 1. Hearing even if using a hearing aid? **(Kodi mumavutika kumva ngakhale mutavala zothandizira kumva?)** | 1. No - no difficulty 2. Yes - some difficulty 3. Yes - a lot of difficulty 4. Cannot hear at all |  |
| - - - 1. Walking or climbing?   **(Kodi mumavutika kuyenda kapena kukwera chitunda?)** | 1. No - no difficulty 2. Yes - some difficulty 3. Yes - a lot of difficulty 4. Cannot walk or climb at all |  |
| - - - 1. Remembering or concentrating?   **( Kodi mumavutika kukumbukira kapena kukhala ndi chidwi pa zinthu?)** | 1. No - no difficulty 2. Yes - some difficulty 3. Yes - a lot of difficulty 4. Cannot remember or concentrate at all |  |
| - - - 1. With (self-care such as) washing all over or dressing?   **(Kodi mumavutika kudzisamalira monga kuzisambitsa kapena kuvala nokha?)** | 1. No - no difficulty 2. Yes - some difficulty 3. Yes - a lot of difficulty 4. Cannot care for myself at all |  |
| - - - 1. Using the usual (customary) language, do you have difficulty communicating/speaking (for example understanding or being understood by others)?   **(Kodi mumavutika kuyakhula, kumva kapena kuti anthu ena akumveni bwino bwino?)** | 1. No - no difficulty 2. Yes - some difficulty 3. Yes - a lot of difficulty 4. Cannot communicate at all |  |

***Instructions to researcher: if the answer to any of the above questions is 2, 3, or 4 ask questions 7 and 8 below. If they answer 1 to all the above questions move to question 9.***

- - - 1. Can you tell me why *you have* such difficulties (*insert cause of the activity limitation as reported above)*? (*NB: make sure you ask the same question about all reported activity limitations.)*

**Mwandifotokozera kuti muli ndi vuto la XX….mungandifotokozereko chimene chinayambitsa? (Remember to ask all the disabilities mentioned in the 6 questions above)**

- - - 1. How long have you had the difficulties that you have identified?

**Kodi vuto limeneli mwakhala nalo kwanthawi yayitali bwanji?**

- - - 1. What does the word disability mean to you?

**Kodi inuyo mukamati munthu olumala mumatanthauza chiyani?**

- - How are people living with disability in your community treated?

**Anthu kunoko amakhala motani ndi anthu olumala?**

- - How do community members support people with disabilities or their households?

**Anthu kunoko amawathandiza motani anthu olumala kapena mabanja awo?**

- - - Who offers support?
      - IHPs, DPOs, CBR, NGO etc

**Ndi ndani yemwe amapereka thandizolo?**

- - - - IHPs, DPOs, CBR, NGO etc
    - How do family members treat people with disabilities within their household?

**Kodi anthu a pabanja pao amamusamalira bwanji munthu olumala?**

- - Is there anyone in your family who considers themselves to be disabled?

**Kodi pa banja panu alipo wina aliyense amene amazitenga kuti ndi olumala?**

- - **If 3 or 4 given as a response to the Washington group questions:** Based on the limitations you described to me earlier would you describe yourselfas having a disability? Why/Why not?

**Mwandifotokozera kuti muli ndi vuto la XX…kodi inuyo mumazitenga kuti ndinu olumala? Fotokozani?**

1. What is your opinion about the general perception and attitude towards people living with disabilities in your community?

**Kodi maganizo anu ndi otani mmene anthu akuno amaonera anthu olumala?**

- 1. How do perceptions vary by:
     1. Type of disability
     2. Sex
     3. Age
     4. Economic Status
     5. Education

**Kodi zimasiyana momwe anthu amawaonera anthu amenewa kutengera ndi:**

- 1. **Wamamuna kapena wa mkazi**
  2. **Mtundu wachilema /kulumala kwake**
  3. **Zaka**
  4. **Kapezedwe ka zinthu (kulemera kapena kusauka)?**
  5. **Maphunziro**

1. What can you say about the participation of people with disability in the community as well as within their household?

**Munganenepo chiyani pa kagwiridwe ntchito kapena kutenga nawo mbali pa ntchito zosiyanasiyana kwa anthu olumala pa banja pawo komanso mmudzi muno? (Ie church/ development household chores/ farming etc**

***Summarise key points from disability discussion.***

***Thank you very much for providing me with information on your understanding of disability. If there is nothing else you would like to add in this area I would like to move on to ask you some questions about your knowledge of chronic cough and lung health.***

### Knowledge of Lung Health

1. When would you call a cough a chronic one?

PROMPT:

- - How long would someone have to have been coughing before you would say it was chronic?

**Kodi chifuwa mumachitcha cha mgonagona patapita nthawi yayitali bwanji? (funsitsani za nthawi yomwe munthuyo wakhala akukhosomola)**

1. What diseases do you know are associated with chronic cough?

PROMPT:

- Asthma, TB, other CAD?

**Kodi ndi matenda anji omwe amagwirizana ndi chifuwa cha mgonagona? Funsitsani za mphumu kapenanso befu (chifuwa chotha phuma)**

1. How would you describe breathlessness or wheezing?

**Kodi befu ndi chiyani?**

1. What diseases do you know that are associated with difficulty in breathing?

PROMPT:

- TB, Asthma, other CAD?

**Kodi ndi matenda anji omwe amagwirizana ndi befu?**

**Funsitsani za mphumu kapenanso befu (chifuwa chotha phuma) (wheezing)**

1. Would you tell us what you know about X? (*Instruction to researcher: X refers to the disease that the ward has previously been diagnosed with or is currently diagnosed with)*

**Mungatiuzeko zomwe mukudziwa za XX (*Instruction to researcher: XX refers to each of the diseases of interest that the respondent is suffering from as mentioned above. You should ask about them in order ie TB, Asthma, CAD.)***

PROMPT:

- What are the causes of these diseases?

**Zomwe zimayambitsa matenda amenewa**

- What are the signs and symptoms?

**Zizindikiro zake ndi zotani?**

- Is this disease associated with shortness of breath?
  - - If yes, how do you tell if someone has shortness of breath?
- **Kodi matenda amenewa amakhudzana ndi befu?**
  - **Mungadziwe bwanji kuti munthu ali ndi befu?**
- How are they transmitted?

**Kufala kwake**

- How are they prevented?

**Kupewa kwake**

- How do you know if someone has this disease?

**Mungadziwe bwanji kuti munthu ali ndi matenda amenewa?**

- What is the treatment for these diseases?

**Mankhwala amatenda amenewa ndi chiyani?**

1. Where did you get the information you have shared with me about X? (*Instruction to researcher: X refers to the disease that the ward has previously been diagnosed with or is currently diagnosed with)*

**Kodi zimene mwafotokozazi mumazimvera kuti?**

PROMPT:

- Within your community, are there groups that provide information about this?
  - Can you tell me who they are? Name them?

**Kodi mdera/mmudzi muno muli magulu/anthu omwe amafalitsa mauthengawa (ie TB, asthma ndi Chronic cough)?**

- **Ndi ndani?**
  - 1. If so, how do they provide this information?
       1. Drama, song, leaflet, talk, poster, radio etc.

**Ngati alipo, amafalitsa motani?**

- - 1. Which group gives the best understanding about this disease(s)? Explain?

**Mwamagulu omwe mwatchulawa ndi magulu ati omwe amafalitsa mauthengawa momveka bwino kwambiri? Fotokozani?**

- - 1. Which method(s) gives a clearer understanding of these disease(s)? Explain?

**Ndi njira ziti zimene zimapereka uthengawu momveka bwino kwambiri? Fotokozani?**

1. What recommendations can you give to improve communication on health information focusing on this disease(s)?

**Munganenepo zotani pofuna kupititsa mtsogolo kafalitsidwe ka mauthenga okhudzana ndi matenda XX – mention the disease? (TB, asthma komanso chifuwa chamgonagona)?**

PROMPT:

- What information would you have liked to have? What would you have liked to know more about?

**Mukanakonda mukanadziwa zotani za matenda amenewa XX- mention the disease?**

- How would you like to receive this information in the future?

**Mungakonde mutamaulandira bwanji uthenga umenewu mtsogolo muno?**

- Are there any specific areas that you would like additional information on?

**Pa matenda amenewa, pali uthenga wina umene mungafune kudziwa?**

***Summarise key points from knowledge discussion.***

***Thank you very much for providing me with information on your knowledge of lung health, unless you want to add anything in this area, I would like to ask you some more questions about how you seek health care when they you get sick.***

### Health Seeking Behaviour for Lung Health

1. Can you tell me the history of how you came to be diagnosed with X?(*Instruction to researcher: X refers to the disease that the ward has previously been diagnosed with or is currently diagnosed with)*

**Mungandiuzeko kuti chinachitika ndi chiyani kuti mufike poti achipatala akupezani ndi XX – mention the disease ie (mphumu, TB kapena chifuwa cha befu)?**

- What symptoms did you experience?
  - - **Kodi munkamva/ kuona zizindikiro zotani?**
- When did you start experiencing these symptoms?
  - - **Mudayamba kudwala liti?**
- What was done?

**Munachita chiyani?**

- - Where did you go to get health care?

**Mudapita kuti?**

- - Why did you go and get health care there?

**Chifukwa chiyani mudasankha kumeneko?**

- - How long was it until you went to get health care after experiencing symptoms?

**Panapita nthawi yayitali bwanji kuchokera pomwe munayamba kuona/ kumva zizindikiro kufikira pomwe munapita kuchipatala kukafuna thandizo la zaumoyo?**

- How did you decide to seek care?

**Mudapanga bwanji chiganizo chokafuna thandizo la zaumoyo?**

- - Who was involved in the decision to seek care?

**Ndi ndani adapanga chiganizo chopita kumeneko?**

- - What happened if there was a disagreement in the decision of where or when to seek care?

**Kodi panali kusagwirizana kwina kulikonse pa za nthawi kapena kokapeza thandizo la zaumoyo pa matenda amenewa- mention the disease?**

**Ngati panali kusagwirizana, zinatha bwanji?**

- If you needed to pay for care, how did you raise the money?

**Ngati panafunikira kulipira, munatani kuti mupeze ndalamazo?**

- - How much money did you use until you got a diagnosis ie transport costs/ food/ accommodation/ medical tests etc ?

**Mudagwiritsa ntchito ndalama zingati kufikira pomwe achipatala adakupezani ndi XX- mention the disease?**

- How were you supported to seek care?

**Mudathandizidwa bwanji kuti mupeze thandizo la za umoyo?**

- - - Who went with you?

**Mudapita ndi ndani?**

- - - Were they male or female?

**Adali amuna kapeza akazi?**

- - - Were they readily available?

**Ankapezeka mosavuta?**

- - - Why did they go with you? What relationship do they have to you?

**Adakuperekezani chifukwa chiyani? Pali ubale wanji?**

- - - What impact does this have on their income/livelihood?

**Kodi ntchito zomwe amagwira okuperekezaniyu zinasokonekera motani?**

- When seeking care how were you treated?

**Kodi mukapita kukafuna thandizo kuchipatala, amakulandirani motani?**

- - What was the attitude/behaviour of medical staff like?

**Nanga azachipatala amakulandirani motani?**

- - If more than one facility accessed; how did this differ between various health care providers?

**Ngati anakapeza thandizo la zaumoyo kumalo osiyanasiyana, panali kusiyana kotani?**

- - - Public/Private; Formal/Informal; Traditional/non-traditional
- How did accessing health care facilities in relation to this illness compare to previous you have had of accessing health care?

**Kutengera pa zimene mwakumana nazo chifukwa cha matenda amenewa – mention the disease- mungasiyanitse bwanji ndi matenda ena? ( attitude/behaviour of health care workers/ treatment costs/ time to get a diagnosis/)**

- - How was it similar?

**Zikufanana bwanji?**

- - How was it different?

**Zikusiyana bwanji?**

1. How are you treated for XX? (*Instruction to researcher: X refers to the disease that the respondent has previously been diagnosed with or is currently diagnosed with)*

**Kodi mumalandira chithandizo chanji cha matenda amenewa?**

- 1. What medication do you receive?
     1. Tablets, injections, inhalers (blue/brown), traditional medicines

**Kodi mumalandira mankhwala anji? (Tablets, injections, inhalers (blue/brown), traditional medicines)**

- 1. Where do you get these medicines from?
     1. Hospital, pharmacy, health facility, traditional healer, IHP
     2. **Kodi chithandizocho mumalandilira kuti? (Hospital, pharmacy, health facility, traditional healer, IHP)**
  2. How often do you travel to get drugs?

**Kodi mumapita mowirikiza bwanji kukatenga mankhwala amenewa kuchipatala?**

- - 1. How far is the journey?

**Kodi ndikutali bwanji?**

- - 1. What are the costs associated with getting more drugs?

**Kodi kupitapita kuchipatala kukatenga mankhwala kwakhudza bwanji moyo wanu wa tsiku ndi tsiku?**

- 1. For how long have you been taking these drugs? (*ask for all the drugs*)

**Kodi mwakhala mukumwa mankhwala amenewa kwanthawi yayitali bwanji?**

- What effects do you think the drugs have on your health?
  - Any social, psychological, economic effects? Explain.

**Mukuganiza kuti kumwa mankhwala amenewa kungakhale ndi zotsatira zotani pa umoyo wanu?**

**Nanga moyo wanu watsiku ndi tsiku (makhalidwe anu ndi anthu/ maganizo/ chuma) wakhudzidwa motani ndi kumwa mankhwala amenewa?**

- Have you experienced any negative side effects of taking any of these drugs?
  - If yes, can you describe them?

**Kodi thupi lanuli likukumana ndi zovuta zanji chifukwa chakumwa mankhala amenewa?**

**Ngati alipo, fotokozani za mavutowo?**

- How well do you adhere to the treatment provided?

**Kodi mumatsatira motani ndondomeko ya kamwedwe ka mankhwala**

- - 1. Have you ever missed drugs?

**Mudayamba mwaphonyapo kumwa mankhwalawa?**

- - 1. How many times?

**Ngati ndi choncho, ndi kangati?**

- - 1. Why?

**Chifukwa chiyani?**

- - How effective do you think the treatment you get from the hospital/other health care providers is?

**Kodi mukuona kuti makhwala omwe mukulandira kuchipatalawo akukuthandizani motani?**

1. What would you say were/are the main challenges you face in accessing health care for X? (*Instruction to researcher: X refers to the disease that the ward has previously been diagnosed with or is currently diagnosed with)*

**Kodi mumakumana ndi mavuto anji pokalandira thandizo la mankhwala amatenda amenewa - mention the disease?**

PROMPT:

- How does this differ between formal and informal health care?

**Kodi zimasiyana bwanji pakati pa kuchipatala ndi thandizo lina ie kwa a sing’aanga?**

- How does this differ between the public and private sector?

**Pali kusiyana pakati pa zipatala za boma ndi zipatala zolipira?**

- What solutions would you suggest to the various challenges you face in accessing health care services?

**Mukuona ngati tingatani pothetsa mavuto amenewa?**

***Summarise key points from health seeking behaviour discussion.***

***Thank you very much for providing me with information on your experience in accessing health care for X* (*Instruction to researcher: X refers to the disease that the respondent has previously been diagnosed with or is currently diagnosed with). Unless you want to add anything in this area, I would like to ask you some final questions about informal health care providers in your community.***

### Perceptions of Informal Health Care Providers (NB: HSAs are not IHPs)

1. What do you understand by the term informal health provider?

**Kodi mumamvetsa kuti mavolontiya a zaumoyo kumudzi ndi ndani?**

1. Can you tell us the different IHPs that are in this community? (*Instruction to the researcher: ask the below in relation to each of the IHPs mentioned.)*

**Kodi ndi magulu ati amavolontiya a za umoyo omwe alipo kudera lino?**

- What services do they offer?

**Amapereka thandizo lanji?**

- - Information (what diseases?), referrals etc.
    - **Pamatenda anji?**
    - **Amatha kutimiza odwala kuchipatala?**
  - Do people use these services? Why/why not?

**Nanga anthu amagwiritsa ntchito upangiri wawo? Fotokozani?**

- What additional services could they provide that they are not already?

**Kupatula ntchito zomwe akugwira mavolontiya a zaumoyo ammudziwa, pali ntchito zina zomwe angagwirenso zokhudzana ndi za umoyo? Fotokozani?**

- What could be done to improve the quality of the services they provide?

**Pachitike chiyani kuti iwowa azipereka thandizo labwino (kolite)?**

1. Who are the target populations of these IHP?

**Kodi ntchito zawo amafikira nazo ndani?**

Are there any groups in your community or neighbouring communities left out of access to IHP services?

**Kodi alipo magulu ena a anthu mdera lino kapena mmadera oyandikana nawo omwe safikiridwa ndi thandizo loperekedwa ndi mavolontiya a zaumoyo amenewa?**

- - If so which are these groups?

**Ngati alipo, ndiati?**

- - Explain?

**Chifukwa chiyani? Fotokozani.**

1. Are there cases in your community where IHPs’ services are shunned? Explain?
   - E.g because of HIV status? Socio-economic status? Educational level? Tribe?

**Kodi zimachitika kuti thandizo lomwe mavolontiya a za umoyo amapereka limakanidwa? Fotokozani.**

**Funsitsani potengera:**

- **maonekedwe,**
- **maphunziro**
- **kupata kwa munthu,**
- **kasunga chinsinsi**
- **mtundu wa munthu**
- **koliteya chithandizo chomwe amapereka**

1. When seeking treatment for X (disease of diagnosis) what interaction did you have with informal health providers?

**Mukamafuna thandizo la matenda amenewa, kodi mumakambirana/kulumikizana ndi mavoluntiya a zaumoyo**

- - What were the advantages and disadvantages of this interaction?

**Mungandifotokozereko za ubwino ndi kuipa kokambirana kapena kulumikizana ndi mavoluntiya a zaumoyo?**

***Summarise key points from IHP discussion.***

***Thank you very much for providing me with information on your knowledge of IHPs and the previous topics we discussed. Is there anything you would like to add regarding any of the topics we have discussed? Many thanks for your time today it has been most useful to our study.***
