# Supplementary material for: Health seeking for chronic lung disease in central Malawi: Adapting existing models using insights from a qualitative study
Source: PLoS One. 2018 Dec 17;13(12):e0208188. doi: 10.1371/journal.pone.0208188 (PMC6296555; doi:10.1371/journal.pone.0208188)
Supplement: S1 Table — (DOC) [file pone.0208188.s002.doc]

**S1 Table: Supplementary Quotes**

| **Theme** | **Supplementary Quotes** |
| --- | --- |
| Understandings and perceptions of chronic lung disease and TB | *‘In my thought this chronic cough is called TB’* (Study site 1, focus group, 40-year-old male) |
| Perceptions of disease severity shaping whether and when to seek care | *‘Another factor (in determining health seeking behaviour) is the condition of the patient. If the situation is severe they will be forced to go even very far’* (Study site 2, interview with health worker in charge at the health centre, 42 year old male)  *‘chronic cough does not attack with force; it starts with a fair condition where one can manage to treat it with medicine from stores, while malaria attacks with full force and people rush to the hospital’* (Study site 1, focus group with health centre committee members, 60 year old male) |
| Perceptions of disease causation shaping where to seek care | *'Some do go for prayers whilst taking the drugs because a person has body and soul, that is what happens, but (we do) not to go to find treatment at the prayers, we go to the prayers after going to the hospital'* (Study site 1, focus group with community men, 35-year-old male)  *‘We believe that when we cough and we have tried taking medicine without seeing any change, after coughing for a long time, one believes that it is someone who has bewitched them’* (Study site 1, focus group with community men, 26-year-old male) |
| Influence of other community members on care seeking | *‘in the past people used to go to traditional healers but because of the awareness campaigns through the HSAs people come to understand that the first thing to do when you are sick is to go to hospital and now this is happening.’* (Study site 2, interview with village chief, 40 year old male) |
| Wider influences on care seeking practices | *‘Others go to buy drugs from the grocery because the hospital is far away, some buy drugs in case they get sick in the middle of the night so they wait to go to the hospital the next day’* (Study site 1, focus group with informal health providers, 40-year-old female)  *‘sometimes sickness comes at a time when you have nothing, so we just ask the owner of the oxcart to help us take the patient to hospital. Thereafter, family members should meet and contribute money to pay for the oxcart’* (Study site 1, focus group with community women, 34 year old female) |
| Availability of healthcare | *‘If the public health facility is experiencing drug stock outs they can just write you the prescription and refer you to the private clinic to buy the medicine’* (Study site 1, interview with the village head, male, age unknown) |
| Diagnosis of lung disease | *‘I started coughing and I went to hospital and they gave me treatment and I went another time also within a short time and I was going to hospital more frequently until the healthy passport book was filled with cough diagnosis’* (Study site 2, interview with a patient with asthma, 33 year old male)  *‘We as health workers mostly deal with what we call public importance diseases such as TB, malaria and HIV, so if we can have the ability to identify diseases like asthma it can be very good and helpful’* (Study site 2, focus group with health surveillance assistants at the health centre, 44 year old male) |
| Communication of lung disease diagnosis | *‘I would like to know if a person receives treatment for asthma is he going to be healed or he will just be protected?’* (Study site 2, focus group with informal health providers at the health centre, 46 year old male) |
| Treatment for chronic lung diseases | *‘us who have asthma we are given aminophylline or salbutamol, Panado and Bactrim and when it is very serious they use the drip’ (*Study site 2, interview with a patient with asthma, 45 year old female)  *‘Facilitator: Why haven’t you ever missed taking the drugs?*  *Repondent: It can be possible that the time I have missed taking the drugs, I can also have an attack, so I take the drugs as a protector so that I can be able to do different chores’* (Study site 2, interview with a patient with asthma, 45 year old female) |
| Experiences of service quality | *‘The traditional healer was very loving because he told me that I should take the girl to hospital first to be examined, then I went to hospital where she diagnosed with asthma’* (Study site 1, focus group with community women, 36 year old female) |
| Perceptions of diagnosis and treatment quality (staff attitudes) | *‘the behaviour also is in that way because of the pressure of work … for one clinician like myself, it is not easy day and night throughout the year and seeing more than 200 people per day, it’s a difficult thing because others will find [you] tired and maybe you will not talk to them in a good mood, they will not have a good picture of you’* (Study site 2, interview with the health worker in charge at the health centre, 42 year old male) |
